# Bariatric surgery and relevant comorbidities: a systematic review and meta-analysis

**DOI:** 10.1007/s00464-025-11528-4

**Published:** 2025-02-07

**Authors:** Claire Wunker, Sunjay Kumar, Peter Hallowell, Amelia Collings, Lindsey Loss, Varun Bansal, Bradley Kushner, Theofano Zoumpou, Tammy Lyn Kindel, D. Wayne Overby, Julietta Chang, Subhashini Ayloo, Andrew F. Sabour, Omar M. Ghanem, Essa Aleassa, Adam Reid, Noe Rodriguez, Ivy N. Haskins, L. Renee Hilton, Bethany J. Slater, Francesco Palazzo

**Affiliations:** 1 Department of Surgery, Saint Louis University, St. Louis, USA; 2 Department of Surgery, Jefferson University, Philadelphia, USA; 3 Department of Surgery, University of Virginia, Charlottesville, USA; 4 Department of Surgery, University of Louisville, Louisville, USA; 5 Department of Surgery, Oregon Health and Science University, Portland, USA; 6 Department of Surgery, University of Colorado, Boulder, USA; 7 Department of Surgery, Washington University in St. Louis, St. Louis, USA; 8 Department of Surgery, Rutgers New Jersey Medical School, Newark, USA; 9 Department of Surgery, Medical College of Wisconsin, Milwaukee, USA; 10 Department of Surgery, University of North Carolina, Chapel Hill, USA; 11 Department of Surgery, Kaiser Permanente Bellevue Medical Center, Bellevue, USA; 12 Department of Surgery, Brown University, Providence, USA; 13 Department of Surgery, University of Nevada, Reno, USA; 14 Department of Surgery, Mayo Clinic, Arizona, USA; 15 Department of Surgery, Cleveland Clinic, Cleveland, USA; 16 Department of Surgery, Southern Illinois University School of Medicine, Springfield, USA; 17 Department of Surgery, University of Nebraska Medical Center, Omaha, USA; 18 Department of Surgery, Medical College of Georgia, Augusta, USA; 19 Department of Surgery, University of Chicago, Chicago, USA; 20 Department of Surgery, Medical Office Building, Thomas Jefferson University Hospital, 1100 Walnut Street, 5 Floor, Philadelphia, PA 19107 USA

**Keywords:** Metabolic surgery, Cholangiogram, IBD, GERD, Bariatric, Comorbidities

## Abstract

**Background:**

Obesity is a growing epidemic in the United States, and with this, has come an increasing volume of metabolic surgery operations. The ideal management of obesity-associated medical conditions surrounding these operations is yet to be determined. This review sought to investigate the routine use of intraoperative cholangiogram (IOC) with cholecystectomy during or after a bypass-type operation, the ideal management of post-sleeve gastrectomy gastroesophageal reflux disease (GERD), and the optimal bariatric operation in patients with known inflammatory bowel disease (IBD).

**Methods:**

Using medical literature databases, searches were performed for randomized controlled trials (RCTs) and non-randomized comparative studies from 1990 to 2022. Each study was screened by two independent reviewers from the SAGES Guidelines Committee for eligibility. Data were extracted while assessing the risk of bias using the Cochrane Risk of Bias 2.0 Tool and the Newcastle–Ottawa Scale for RCTs and cohort studies, respectively. A meta-analysis was performed using random effects.

**Results:**

Routine use of IOC was associated with a significantly decreased rate of common bile duct injury and a trend towards decreased intraoperative complications, perioperative complications, and mortality. The rates of reoperation, postoperative pancreatitis, cholangitis, and choledocholithiasis were low in the routine use of the IOC group, but no non-routine use studies evaluated these outcomes. After sleeve gastrectomy, GERD-specific quality of life was significantly higher in the surgically treated group compared to the medically treated group. Bypass-type operations had worse outcomes of IBD sequelae than sleeve gastrectomy, including pain, patient perception, and fistula formation. Sleeve patients had lower mortality and fewer short- and long-term complications.

**Conclusions:**

Low-quality data limited the conclusions that were drawn; however, trends were observed favoring the routine use of IOC during cholecystectomy for patients with bypass-type anatomy, surgical treatment of GERD post-sleeve gastrectomy, and sleeve gastrectomy in IBD patients. Future research proposals are suggested to further answer the questions posed.

**Supplementary Information:**

The online version contains supplementary material available at 10.1007/s00464-025-11528-4.

## Introduction

Almost 42% percent of American patients have obesity [1]. As this epidemic continues, metabolic surgery procedures are becoming more common [2], and thus, bariatric surgery’s impact on the care of other obesity-associated medical conditions is an important consideration both pre- and postoperatively. This review sought to evaluate three obesity-associated medical conditions that can impact the care of patients with obesity who have undergone or are being evaluated for weight loss surgery: cholelithiasis, gastroesophageal reflux disease (GERD), and inflammatory bowel disease (IBD).

Patients who undergo metabolic surgery procedures have an increased incidence of gallstones, up to 25%, compared to 10–20% in the general population [3–5]. When these patients develop choledocholithiasis, the altered anatomy typically precludes endoscopic retrograde cholangiopancreatography (ERCP), and alternative treatment strategies must be pursued. We sought to determine if bariatric patients with bypass-type anatomy should undergo routine intraoperative cholangiogram at the time of cholecystectomy.

Approximately half of all patients with obesity suffer from concurrent GERD, and sleeve gastrectomy has been shown to worsen or promote the development of GERD [6]. For this reason, Roux-en-Y gastric bypass (RYGB) is often the operation of choice in patients with obesity and GERD. However, little is known about the optimal treatment of worsening or de novo GERD post-sleeve gastrectomy. We investigated whether medical treatment with proton pump inhibitors or surgical treatment, including conversion to an RYGB, leads to superior outcomes in these patients.

The final medical condition we investigated in conjunction with bariatric surgery was inflammatory bowel disease (IBD). While only 0.7% of the US population is affected, 15–40% of IBD patients also have obesity [7, 8]. Pre-operative assessment of IBD patients is essential when considering which bariatric procedure to perform. The bariatric principle of bypassing the intestine in a malabsorptive operation is at odds with the goal of preserving as much intestine as possible in a patient with IBD [9]. For this reason, we aimed to determine if sleeve gastrectomy was superior to intestinal bypass in patients with both obesity and IBD.

## Methods and materials

A working group was formed from members of the Society of American Gastrointestinal and Endoscopic Surgeons (SAGES) Guidelines Committee. This group received formal training in systematic review methodology consistent with the Preferred Reporting Items for Systematic Reviews and Meta-Analysis (PRISMA) guidelines [10]. Three key questions (KQs) were devised using the Population, Intervention, Comparator, Outcomes (PICO) format.

- KQ 1) Should routine IOC or no-routine IOC be used for patients undergoing cholecystectomy during or after gastrointestinal bypass-type bariatric procedures (RYGB, duodenal switch (DS) and others)? Outcomes included postoperative choledocholithiasis, intraoperative complications, perioperative complications, long-term postoperative complications, mortality, ERCP, reoperation, postoperative pancreatitis, postoperative cholangitis, common bile duct (CBD) injury, percutaneous transhepatic cholangiography, cost, and quality of life. 30-day perioperative complications included grade 2 or greater on the Clavien-Dindo scale. Long-term complications were those occurring greater than 30 days postoperatively.
- KQ 2) Should surgical or medical therapy be used for GERD after laparoscopic sleeve gastrectomy? Outcomes included DeMeester score, impedance score, acid exposure time, mean number of reflux episodes in 24 h, mean number of endoscopies in follow-up, Barrett’s esophagus presence, esophagitis presence, patient-reported long-term symptom control, overall quality of life, GERD-specific quality of life, drug-related serious adverse events, complications, and mortality. The patient-reported long-term symptom control was measured at least 6 months postoperatively.
- KQ 3) Should sleeve gastrectomy or intestinal bypass-type procedures be used in patients who have concurrent obesity and IBD. Outcomes included worsening pain, worsening of other IBD symptoms (obstruction, bleeding, fistula, perforation), ulceration, overall quality of life, gastrointestinal (GI)-specific quality of life, patient-reported worsening of IBD, drug-related serious adverse events, perioperative complications, long-term postoperative complications (> 30d), mortality, and reoperation. Perioperative complications again included those occurring up to 30 days postoperatively and grade 2 or greater on the Clavien-Dindo scale. Long-term complications were defined as those occurring greater than 30 days postoperatively.

### Included studies

Search criteria included English language, randomized controlled trials, and observational studies, including single-arm studies, from 1990 to 2022. This timeframe was chosen to encompass the available literature on bariatric procedures. Individual case reports were excluded, while case series were included. Non-English articles and studies with only abstracts available were also excluded.

### Included participants

All KQs addressed the adult population only.

- KQ1 included patients with a prior bypass-type operation who were undergoing cholecystectomy. Bypass-type procedures included RYGB, one anastomosis gastric bypass (OAGB), duodenal switch (DS), and single anastomosis duodeno-ileal bypass (SADI).
- KQ2 included patients who had been diagnosed with GERD and had undergone sleeve gastrectomy.
- KQ3 included patients with both IBD and obesity who were undergoing bariatric surgery. Patients with ulcerative colitis and Crohn’s disease were both included.

### Interventions

In KQ1, the intervention of routine intraoperative cholangiography during cholecystectomy was compared against not performing routine intraoperative cholangiography during cholecystectomy.

In KQ2, all interventional treatments for reflux, including conversion to RYGB, hiatal hernia repair, magnetic sphincter augmentation (MSA), Stretta, fundoplication, fundectomy, falciform ligament wrap, transoral incisionless fundoplication, and other less commonly used surgical techniques, were compared against medical therapy, including both proton pump inhibitors (PPIs) and H2 blockers.

In KQ3, the intervention was sleeve gastrectomy, which was compared against any type of intestinal bypass operation for weight loss, including RYGB, OAGB, DS, and SADI.

### Search strategies

The working group devised search strategies for each KQ with the assistance of a medical librarian. PubMed, Embase, Cochrane, and clinicaltrials.gov were searched. Our search parameters were limited to January 1990–March 2022. The results collected were combined and then exported to Covidence (Veritas Health Innovation, Melbourne, Australia) for further screening and extraction. Full search strategies for each KQ are available in Online Appendix 1.

### Study selection

The working group underwent calibration of study selection using Abstrackr (Brown University, Providence, Rhode Island, US). The standard operating procedure followed has been outlined previously [11]. After completing this step, the studies were uploaded to Covidence and screened, first at the title and abstract level and then at the full-text level. Two reviewers screened each paper at both phases. Disagreements in screening were resolved through consensus, and if consensus was not achieved, then a third reviewer was utilized.

### Data extraction and management

Data were extracted by two members of the working group using a standardized extraction form that had been imported into Covidence. The data included study characteristics, patient demographics, details of the interventions, and outcomes. Consensus was obtained between reviewers through discussion. The authors were not contacted for missing study data. The data were then exported from Covidence and reviewed for accuracy and consistency.

#### Assessment of bias

Each study was evaluated by two reviewers to assess bias. The Cochrane Risk of Bias 2.0 Tool was used for RCTs, and the modified Newcastle–Ottawa Scale (NOS) was used for non-randomized studies. Discrepancies between the two reviewers were discussed, and a third reviewer would act as a tiebreaker if unable to reach a consensus.

#### Data analysis

RevMan (Version 5.3.5) was used to perform meta-analysis using a Mantel–Haenszel random-effects model. Risk ratios (RR) and odds ratios (OR) were calculated for dichotomous outcomes from randomized and non-randomized studies, respectively. Inverse variance-weighted mean difference for continuous outcomes was utilized. For continuous outcomes using multiple scales, a standardized mean difference (SMD) was used. Heterogeneity between studies was assessed using measures of *I*^*2*^ and *χ*^*2*^. All comparative studies, including observational and high risk of bias, are presented, but results and conclusions focus on randomized controlled trials and low risk of bias studies when available.

## Results

### Question 1) Should routine IOC or no IOC be used for patients undergoing cholecystectomy during or after gastrointestinal bypass-type bariatric procedures (RYGB and DS, etc.)?

**Fig. 1:**
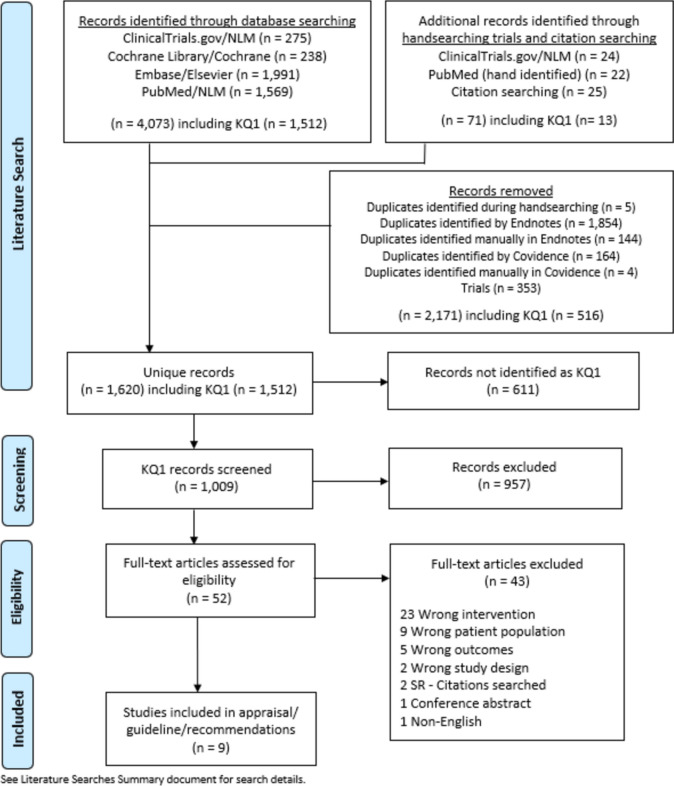
PRISMA diagram for KQ 1

**Fig. 2:**
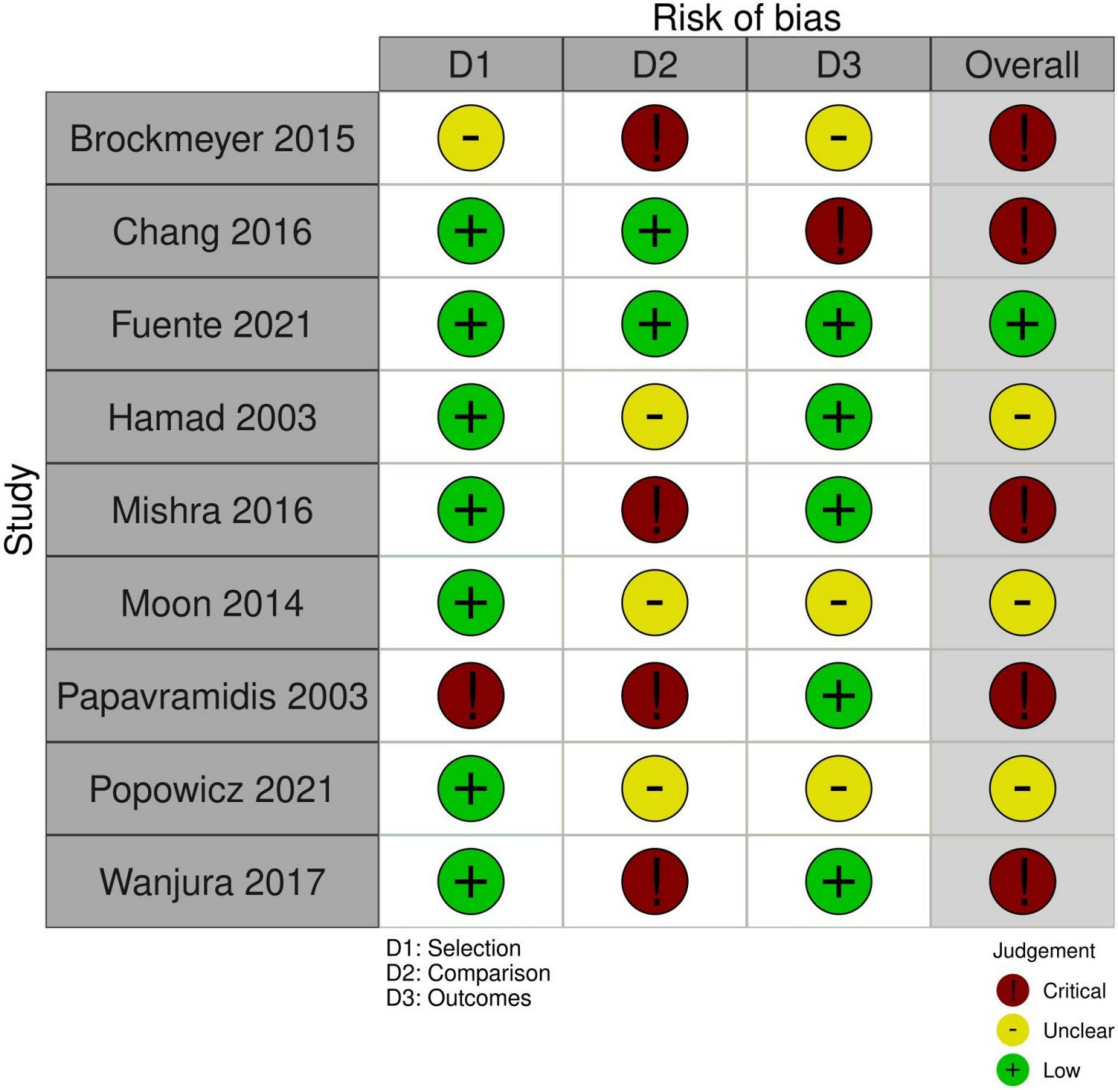
Stoplight chart of risk of bias for the included studies

A total of 1068 publications were identified during the literature search, resulting in 1009 publications after removing duplicates. These were then screened by their title and abstract, further reducing that number to 52, which were then further assessed at the full-text level for data extraction. Data were extracted from 9 articles, which were all included in the final analysis (Table 1) [12–20]. The PRISMA flow diagram for the systematic review is seen in Fig. 1. There were no randomized controlled trials for KQ1, and only one study directly compared routine and non-routine use of IOC. The results can be found in Table 2 (routine use of IOC) and Table 3 (non-routine use of IOC). The risk of bias assessment for the included studies is in Fig. 2.

**Table 1:** Summary of included studies for KQ1

First Author Last Name	Year of publication	Funding source	Study design	Number of routine IOC	Number of no-routine IOC
Brockmeyer [12]	2015	None specified	Single-center retrospective cohort	88	0
Chang [13]	2016	None specified	Retrospective cohort	45	89
Fuente [14]	2021	None specified	Single-center retrospective cohort	57	0
Hamad [15]	2003	None specified	Retrospective cohort	0	94
Mishra [16]	2016	None specified	Single-center retrospective cohort	0	6
Moon [17]	2014	Ethicon Endosurgery	Retrospective cohort	367	0
Papavramidis [18]	2003	None specified	Single-center prospective cohort	0	34
Popowicz [19]	2021	Grant from Rutoch Rickard Julins Research Foundation	Multicenter retrospective cohort	55	0
Wanjura [20]	2017	Örebro University Research Committee (Grant number OLL-488991), Olle Engqvist Research Foundation (no grant number given)	Multicenter retrospective cohort	1165	0

**Table 2:** Outcomes data for the routine use of IOC in post-bariatric surgery patients

Study name	Event rate (%)	Lower limit (%)	Upper limit (%)	*I*^*2*^ (%)
*CBD Injury*	*0.40*	*0.17*	*0.97*	*0.00*
Brockmeyer 2015	0.56	0.04	8.34	
Wanjura 2017	0.35	0.13	0.94	
Fuente 2021	0.86	0.05	12.33	
*ERCP or Laparoscopic assisted required intraoperatively*	3.73	1.74	7.82	0.00
Brockmeyer 2015	4.55	1.72	11.49	
Fuente 2021	0.86	0.05	12.33	
Popowicz 2021	3.64	0.91	13.41	
*ERCP (or lap-assisted ERCP) required—Postoperative*	*0.95*	*0.53*	*1.68*	*0.00*
Brockmeyer 2015	2.27	0.57	8.63	
Wanjura 2017	0.71	0.35	1.40	
Fuente 2021	0.86	0.05	12.33	
Popowicz 2021	1.82	0.26	11.81	
*Intraoperative complications (total)*	*2.72*	*0.79*	*8.96*	*0.00*
Fuente 2021	0.86	0.05	12.33	
Popowicz 2021	3.64	0.91	13.41	
*Mortality*	*0.60*	*0.19*	*1.84*	*0.00*
Brockmeyer 2015	0.56	0.04	8.34	
Wanjura 2017	0.04	0.00	0.68	
Fuente 2021	0.86	0.05	12.33	
Popowicz 2021	0.89	0.06	12.73	
Moon 2014	2.27	0.14	27.74	
Chang 2016	1.09	0.07	15.14	
*Perioperative complications (*< *30d)*	*10.60*	*5.93*	*18.24*	*55.20*
Wanjura 2017	13.76	11.87	15.89	
Fuente 2021	3.51	0.88	12.97	
Popowicz 2021	10.91	4.98	22.23	
*Postoperative cholangitis*	*0.31*	*0.11*	*0.89*	*0.00*
Fuente 2021	0.86	0.05	12.33	
Wanjura 2017	0.26	0.09	0.82	
*Postoperative choledocholithiasis*	*0.56*	*0.21*	*1.49*	*13.20*
Wanjura 2017	0.35	0.13	0.94	
Fuente 2021	0.86	0.05	12.33	
Popowicz 2021	1.82	0.26	11.81	
*Postoperative complications (long-term)*	*0.86*	*0.05*	*12.33*	*0.00*
Fuente 2021	0.86	0.05	12.33	
*Postoperative pancreatitis*	*0.75*	*0.40*	*1.41*	*0.00*
Brockmeyer 2015	1.14	0.16	7.62	
Wanjura 2017	0.62	0.29	1.29	
Fuente 2021	0.86	0.05	12.33	
Popowicz 2021	1.82	0.26	11.81	
*Reoperation—laparoscopic or open common bile duct exploration*	*3.87*	*2.93*	*5.10*	*0.00*
Wanjura 2017	3.97	2.98	5.27	
Fuente 2021	0.86	0.05	12.33	
Popowicz 2021	3.64	0.91	13.41	
Moon 2014	2.27	0.14	27.74	

Italics indicate outcomes and cumulative event rates

**Table 3:** Outcome data in non-routine IOC use﻿

Study name	Event rate (%)	Lower limit (%)	Upper limit (%)	*I*^2^ (%)
*CBD injury*	*0.53*	*0.03*	*7.85*	*0.00*
Hamad 2003	0.53	0.03	7.85	
*ERCP (or lap-assisted ERCP) required—Intraoperative*	*12.12*	*4.62*	*28.18*	*0.00*
Mishra 2016	12.12	4.62	28.18	
*Intraoperative complications (total)*	*14.71*	*6.26*	*30.82*	*0.00*
Papavramidis 2003	14.71	6.26	30.82	
*Mortality*	*1.04*	*0.30*	*3.52*	*0.00*
Hamad 2003	1.06	0.15	7.16	
Mishra 2016	1.47	0.09	19.59	
Papavramidis 2003	1.43	0.09	19.12	
Chang 2016	0.51	0.03	7.63	
*Perioperative complications (*< *30d) Clavien dindo*	*18.06*	*12.30*	*25.72*	*0.00*
Hamad 2003	19.15	12.41	28.36	
Papavramidis 2003	14.71	6.26	30.82	

Italics indicate outcomes and cumulative event rates

### CBD injury

Three single-arm studies used routine IOC and evaluated CBD injury, while only a single study evaluated CBD injury without routine IOC. In the routine IOC group, the cumulative rate of injury was 0.40% (CI 0.17–0.97%, *I*^2^ = 0) compared to the single study that evaluated CBD injury without routine use of IOC with an event rate of 0.53% (CI 0.03–7.85%) which was not statistically significant.

### ERCP required intraoperatively

There were four observational studies that categorized whether an ERCP was required intraoperatively. In the routine IOC group, the cumulative event rate was 3.73% (CI 1.74–7.82%, *I*^2^ = 0). In Mishra et al. (2016), ERCP intraoperatively without routine use of IOC had an event rate of 12.12% (CI 4.62–28.18%); however, this was not statistically significant.

### Intraoperative complications

There were three single-arm studies (two with routine use of IOC and one without routine use of IOC) that evaluated intraoperative complications. The two routine use of IOC studies had an event rate of 2.72% (CI 0.79–8.96%, *I*^2^ = 0.00%), while the single study without routine use of IOC had an event rate of 14.71% (CI 6.26–30.82%); however, this was not statistically significant.

### Mortality

Mortality was the most consistently reported outcome but did not achieve statistical significance. Nine studies evaluated mortality. The cumulative event rate in the routine IOC studies was 0.6% (CI 0.19–1.84%, *I*^2^ = 0.00%). The event rate in the non-routine IOC use group was 1.04% (CI 0.30–3.52%, *I*^2^ = 0.00%). There was one comparative study by Chang et al. (2016) where mortality without routine IOC was 0.51% (95% CI 0.03–7.63%) and 1.09% (95% CI 0.07–15.14%) with routine use.

### Perioperative complications

There were five single-arm studies that evaluated perioperative complications within 30 days. In the studies that evaluated no-routine use of IOC, the event rate was 18.06% (CI 12.30–25.72%, *I*^2^ = 0.00%), which was higher than routine use of IOC where the event rate was 10.6% (CI 5.93–18.24%, *I*^2^ = 55.20%) but not to a significant level. In addition, the *I*^2^ value for the routine use studies was 55.20%.

### Remaining outcomes

Of note, during our data extraction, there were several outcomes for which data were available for the routine use of the IOC group but not for the non-routine use of the IOC group. This meant that comparison was not possible. The data for the routine use of the IOC group are presented in Table 2. The outcomes and number of studies that reported each outcome in which comparison was not possible included ERCP postoperatively (4 studies, event rate 0.95%, 95% CI 0.53–1.68%, *I*^2^ = 0.00), postoperative cholangitis (2 studies, event rate 0.31%, 95%CI 0.11–0.89%, *I*^2^ = 0.00), postoperative choledocholithiasis (3 studies, event rate 0.56%, 95% CI 0.21–1.49%, *I*^2^ = 13.20), postoperative complications over 30 days (1 study, event rate 0.86%, 95% CI 0.05–12.33%), postoperative pancreatitis (4 studies, event rate 0.75%, 95% CI 0.40–1.41%, *I*^2^ = 0.00), and reoperation (4 studies, event rate 3.87%, 95%CI 2.93–5.10%, *I*^2^ = 0.00).

### Question 2) Should surgical versus medical therapy be used for GERD status post Laparoscopic Sleeve Gastrectomy (LSG)?

**Fig. 3:**
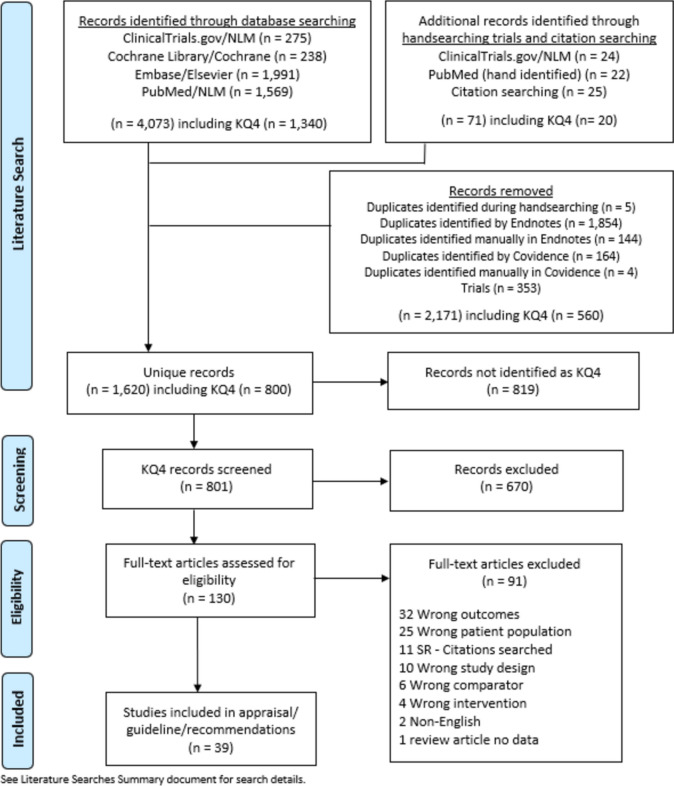
PRISMA diagram for KQ 2

**Fig. 4:**
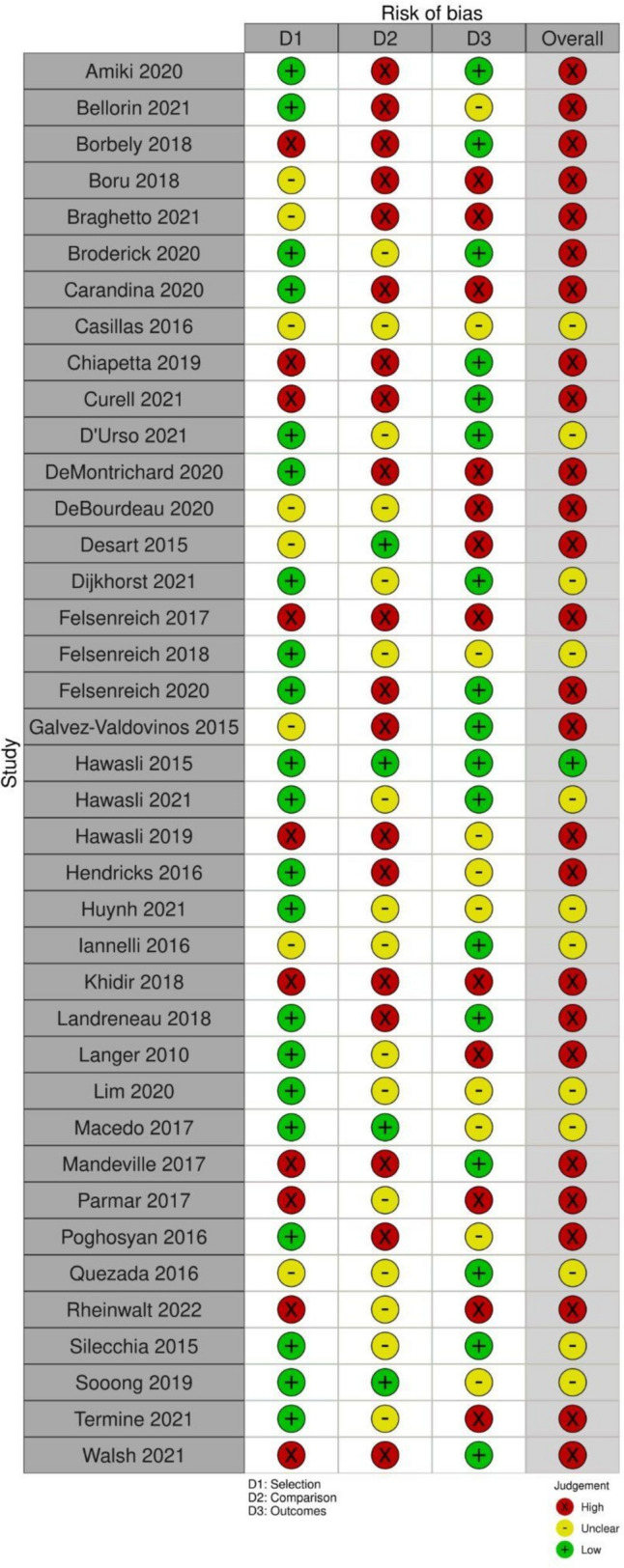
Stoplight diagram for studies included in KQ2

A literature search was completed for KQ2. Using the databases mentioned, 844 publications were identified during the literature search. After accounting for duplicates, 800 articles were screened by title and abstract. This yielded 130 articles for full-text screening and then 39 for data extraction. Thirty-nine articles were included in the meta-analysis (Table 4) [21–59]. The PRISMA flow diagram for the systematic review is seen in Fig. 3. The risk of bias assessment for the included studies is in Fig. 4. The results from the data extraction are in Tables 5, 6, 7, and 8.

**Table 4:** All included studies for KQ2

First Author Last Name	Year of publication	Funding source	Study design	Surgical intervention	Number of surgically treated patients	Number of medically treated patients
Amiki [21]	2020	Departmental resources	Single-center retrospective cohort	Roux-en-Y gastric bypass	9	0
Bellorin [22]	2021	Internally funded	Single–center prospective cohort	Roux-en-Y gastric bypass or Magnetic sphincter augmentation	33	0
Borbély [23]	2018	None specified	Multicenter prospective cohort	Lower esophageal sphincter stimulation	17	0
Boru [24]	2018	None specified	Multicenter Retrospective Cohort	Roux-en-Y gastric bypass	30	0
Braghetto [25]	2021	None specified	Single-center retrospective cohort	Roux-en-Y gastric bypass	39	205
Broderick [26]	2020	None specified	Multicenter retrospective cohort	Magnetic sphincter augmentation	13	0
Carandina [27]	2020	None specified	Retrospective cohort	Roux-en-Y gastric bypass	80	0
Casillas [28]	2016	None specified	Single-center retrospective cohort	Roux-en-Y gastric bypass	48	0
Chiappetta [29]	2019	None specified	Multicenter prospective cohort	Roux-en-Y gastric bypass (*N* = 21), One anastomosis gastric bypass(*N* = 34)	55	0
Curell [30]	2021	None specified	Single-center retrospective cohort	Roux-en-Y gastric bypass	35	0
Debourdeau [31]	2020	None specified	Multicenter retrospective cohort	Anti-reflux mucosectomy	6	0
De Montrichard [32]	2020	None specified	Retrospective cohort	Roux-en-Y gastric bypass	36	0
Desart [33]	2015	None specified	Single-center retrospective cohort	Magnetic sphincter augmentation	7	0
Dijkhorst [34]	2021	None specified	Multicenter prospective cohort	Roux-en-Y gastric bypass	12	0
D'Urso [35]	2021	IHU Strasbourg	Single-center retrospective cohort	Roux-en-Y gastric bypass	60	0
Felsenreich [36]	2017	None specified	Multicenter retrospective cohort	Roux-en-Y gastric bypass	0	10
Felsenreich[37]	2018	None specified	Multicenter retrospective cohort	Roux-en-Y gastric bypass (one duodenal switch, one Santoro procedure)	25	0
Felsenreich[38]	2020	Medical University of Vienna, Steffi Rothe	Single-center retrospective cohort	Roux-en-Y gastric bypass	10	0
Gálvez-Valdovinos [39]	2015	None specified	Single-center retrospective cohort	Hiatal hernia repair with cardiopexy	15	0
Hawasli [40]	2015	None specified	Single-center retrospective cohort	Anterior fundoplication, imbrication of fundus if dilated, re-sleeve if fundus and body of sleeve dilated	6	0
Hawasli [41]	2019	None specified	Single-center retrospective cohort	Magnetic sphincter augmentation	13	0
Hawasli [42]	2021	None specified	Multicenter retrospective cohort	Ligamentum teres cardiopexy	10	0
Hendricks [43]	2016	None specified	Single-center retrospective cohort	Roux-en-Y gastric bypass	4	36
Huynh [44]	2021	None specified	Single-center retrospective cohort	Roux-en-Y gastric bypass	41	0
Iannelli [45]	2016	None specified	Single-center retrospective cohort	Roux-en-Y gastric bypass	11	0
Khidir [46]	2018	None specified	Single-center retrospective cohort	Stretta procedure	15	0
Landreneau [47]	2018	None specified	Single-center retrospective cohort	Roux-en-Y gastric bypass	89	0
Langer [48]	2010	None specified	Single-center retrospective cohort	Roux-en-Y gastric bypass	3	0
Lim [49]	2020	None specified	Single-center retrospective cohort	Roux-en-Y gastric bypass	14	0
Macedo [50]	2017	None specified	Single-center retrospective cohort	Hiatal hernia repair with cruroplasty	9	0
Mandeville [51]	2017	None specified	Single-center retrospective cohort	Roux-en-Y gastric bypass	26	0
Parmar [52]	2017	None specified	Retrospective cohort	Roux-en-Y gastric bypass	10	0
Poghosyan [53]	2016	None specified	Two-center retrospective cohort	Roux-en-Y gastric bypass	34	0
Quezada [54]	2016	None specified	Single-center retrospective cohort	Roux-en-Y gastric bypass	16	0
Rheinwalt [55]	2022	None specified	Retrospective cohort	Roux-en-Y gastric bypass or OAGB	108	0
Silecchia [56]	2015	None Specified	Retrospective cohort	Fundectomy	19	0
Soong [57]	2019	None specified	Single-center retrospective cohort	Hiatal hernia repair	28	0
Termine [58]	2021	None specified	Multicenter retrospective cohort	Roux-en-Y gastric bypass	94	0
Walsh [59]	2021	None specified	Single-center prospective cohort	Endoscopic resection and plication technique	11	0

**Table 5:** Continuous outcomes for medical treatments in KQ2

Study name	Mean	Lower limit	Upper limit	I^2^	N	Medical treatment received
*Acid exposure time (total)*	*9.70*	*6.15*	*13.25*	*0.00%*		
Felsenreich 2018	9.70	5.29	14.11		16	
*DeMeester score*	*44.10*	*27.96*	*60.24*	*0.00%*		
Felsenreich 2018	44.10	27.96	60.24		19	
*Mean # of reflux episodes in 24 h*	*63.40*	*33.15*	*93.65*	*0.00%*		
Felsenreich 2017	63.40	33.15	93.65		10	
*Quality of Life, GERD specific—continuous 10-year RSI mean*	*8.40*	*3.81*	*12.99*	*0.00%*		
Felsenreich 2017	8.40	3.81	12.99		10	

Italics indicate outcomes and cumulative event rates

**Table 6:** Continuous variables assessed for surgical treatments

Study name	Mean	Lower limit	Upper limit	I^2^	Surgical treatment
*Acid exposure time (total)*	*3.80*	*0.79*	*6.81*	*0%*	
Felsenreich 2020	3.80	0.79	6.81		Roux-en-Y bypass
*DeMeester score*	*30.52*	*1.35*	*59.69*	*90.38%*	
Felsenreich 2020	16.30	7.02	25.58		Roux-en-Y bypass
Carandina 2020	46.10	30.54	61.66		Roux-en-Y bypass
*Impedance score post-intervention mean*	*16.30*	*13.62*	*18.98*	*0%*	
Felsenreich 2020	16.30	13.62	18.98		Roux-en-Y bypass
*Mean # of reflux episodes in 24 h*	*49.00*	*26.13*	*71.87*	*0%*	
Felsenreich 2020	49.00	26.13	71.87		Roux-en-Y bypass
*Quality of Life, GERD specific (Gastro-Intestinal Quality of Life Index)*	*113.50*	*98.19*	*128.81*	*0%*	
Felsenreich 2020	113.50	98.19	128.81		Roux-en-Y bypass
*Quality of Life, GERD specific (GERD-health-related quality of life)*	*7.29*	*4.38*	*10.20*	*0%*	
Huynh 2021	7.29	4.38	10.20		Roux-en-Y bypass
*Quality of Life, GERD specific (Visick Score)*	*1.22*	*0.97*	*1.47*	*0%*	
Boru 2018	1.22	0.97	1.47		Roux-en-Y bypass
*Quality of Life, GERD specific (Health-Related Quality of Life)*	*9.06*	*4.80*	*13.33*	*0%*	
Broderick 2020	8.00	3.02	12.98		MSA
Hawasli 2019	12.00	3.73	20.27		MSA
*Quality of Life, GERD specific (GERD-health-related quality of life)*	*27.20*	*-1.30*	*55.70*	*98.49%*	
Khidir 2018	41.80	36.23	47.37		Stretta
Walsh 2021	12.72	8.47	16.97		Endoscopic resection with plication
*Quality of Life, GERD specific—continuous 1 month (GERD-health-related quality of life)*	*12.30*	*7.83*	*16.77*	*0%*	
Soong 2019	12.30	7.83	16.77		Hiatal repair with gastropexy
*Quality of Life, GERD specific—continuous 12 months (GERD-health-related quality of life)*	*17.40*	*9.56*	*25.24*	*0%*	
Soong 2019	17.40	9.56	25.24		Hiatal repair with gastropexy
*Quality of Life, GERD specific—continuous 6 month (GERD-health-related quality of life)*	*16.80*	*10.47*	*23.13*	*0%*	
Soong 2019	16.80	10.47	23.13		Hiatal repair with gastropexy
*Quality of Life, overall (Gastro-Intestinal Quality of Life Index)*	*5.10*	*3.64*	*6.56*	*0%*	
Felsenreich 2020	5.10	3.64	6.56		Roux-en-Y bypass
*Quality of Life, overall (GERD-health-related quality of life)*	*41.80*	*36.23*	*47.37*	*0%*	
Khidir 2018	41.80	36.23	47.37		Stretta
*Quality of Life, overall (GERD score questionnaire)*	*16.06*	*8.34*	*23.78*	*0%*	
Desart 2015	5.14	4.86	5.42		MSA

Italics indicate outcomes and cumulative event rates

**Table 7:** Binary outcomes with medical treatment

Study name	Event rate	Lower limit	Upper limit	*I*^2^		Medical treatment received
*Barrett's Esophagus*	*8.41%*	*2.50%*	*24.79%*	*76.61%*	*230*	
Braghetto 2021	4.88%	2.64%	8.83%		205	PPI
Felsenreich 2018	16.00%	6.14%	35.69%		25	Not specified
*Complications (leak, DVT, blood loss, marginal ulcer, internal hernia, Endoscopic Mucosal injury, carcinoma *in situ*, or adenocarcinoma)*	*0.24%*	*0.02%*	*3.76%*	*0.00%*	*205*	
Braghetto 2021	0.24%	0.02%	3.76%		205	PPI
*Esophagitis present (Grade A, B, or C)*	*27.41%*	*22.03%*	*33.54%*	*0.00%*	*230*	
Braghetto 2021	27.80%	22.10%	34.33%		205	PPI
Felsenreich 2018	24.00%	11.20%	44.16%		25	Not specified
*Long-term symptom control (patient reported;* > *6 months)*	*71.54%*	*48.96%*	*86.82%*	*85.83%*	*287*	
Braghetto 2021	80.98%	75.02%	85.78%		205	PPI
Hawasli 2019	54.55%	26.81%	79.72%		11	PPI
Hendricks 2016	88.89%	73.89%	95.77%		36	PPI
Termine 2021	48.57%	32.74%	64.70%		35	PPI

Italics indicate outcomes and cumulative event rates

**Table 8:** Binary outcomes of surgical treatments

Study name	Event rate	Lower limit	Upper limit	*I*^2^	Roux-en-Y Bypass, MSA or Other surgical or endoscopic interventions	Other treatment specification
*Barrett's esophagus*	*20.00%*	*5.04%*	*54.07%*	*0.00%*		
Felsenreich 2020	20.00%	5.04%	54.07%		Roux-en-Y bypass	
*Complications (leak, DVT, blood loss, marginal ulcer, internal hernia, Endoscopic Mucosal injury, carcinoma *in situ*, or adenocarcinoma)*	*15.21%*	*11.73%*	*19.49%*	*6.88%*		
Bellorin 2021	15.00%	4.92%	37.58%		Roux-en-Y bypass	
Boru 2018	16.67%	5.47%	40.86%		Roux-en-Y bypass	
Braghetto 2021	10.26%	3.90%	24.33%		Roux-en-Y bypass	
Carandina 2020	8.75%	4.23%	17.24%		Roux-en-Y bypass	
Chiappetta 2019	9.52%	2.39%	31.13%		Roux-en-Y bypass	
Curell 2021	5.71%	1.43%	20.16%		Roux-en-Y bypass	
D'Urso 2021	23.33%	14.33%	35.63%		Roux-en-Y bypass	
Huynh 2021	17.07%	8.36%	31.71%		Roux-en-Y bypass	
Iannelli 2016	17.50%	8.58%	32.41%		Roux-en-Y bypass	
Lim 2020	28.57%	11.15%	56.05%		Roux-en-Y bypass	
Parmar 2017	10.00%	1.39%	46.72%		Roux-en-Y bypass	
Quezada 2016	18.75%	6.17%	44.75%		Roux-en-Y bypass	
Termine 2021	3.12%	0.19%	35.03%		Roux-en-Y bypass	
*Esophagitis Present (Grade A, B, or C)*	*15.00%*	*6.42%*	*31.21%*	*62.41%*		
Felsenreich 2020	20.00%	5.04%	54.07%		Roux-en-Y bypass	
Carandina 2020	34.78%	18.44%	55.71%		Roux-en-Y bypass	
Curell 2021	5.71%	1.43%	20.16%		Roux-en-Y bypass	
Lim 2020	6.25%	0.38%	53.86%		Roux-en-Y bypass	
Quezada 2016	6.25%	0.87%	33.54%		Roux-en-Y bypass	
Amiki 2020	50.00%	16.79%	83.21%		Roux-en-Y bypass	
De Montrichard 2020	5.56%	1.39%	19.67%		Roux-en-Y bypass	
*Long-term symptom control (patient reported;* > *6 months)*	*80.05%*	*73.77%*	*85.13%*	*37.17%*		
Bellorin 2021	85.00%	62.42%	95.08%		Roux-en-Y bypass	
Boru 2018	83.33%	59.14%	94.53%		Roux-en-Y bypass	
Carandina 2020	71.25%	60.43%	80.09%		Roux-en-Y bypass	
Curell 2021	74.29%	57.51%	86.04%		Roux-en-Y bypass	
D'Urso 2021	98.08%	75.64%	99.88%		Roux-en-Y bypass	
Huynh 2021	62.50%	44.90%	77.32%		Roux-en-Y bypass	
Iannelli 2016	95.83%	57.54%	99.74%		Roux-en-Y bypass	
Lim 2020	92.86%	62.97%	99.00%		Roux-en-Y bypass	
Parmar 2017	80.00%	45.93%	94.96%		Roux-en-Y bypass	
Quezada 2016	62.50%	37.72%	82.10%		Roux-en-Y bypass	
Termine 2021	86.67%	59.46%	96.64%		Roux-en-Y bypass	
Amiki 2020	87.50%	46.27%	98.27%		Roux-en-Y bypass	
Casillas 2016	96.88%	80.89%	99.56%		Roux-en-Y bypass	
Dijkhorst 2021	75.00%	44.82%	91.72%		Roux-en-Y bypass	
Felsenreich 2018	95.83%	57.54%	99.74%		Roux-en-Y bypass	
Hendricks 2016	75.00%	23.78%	96.65%		Roux-en-Y bypass	
Landreneau 2018	75.00%	44.82%	91.72%		Roux-en-Y bypass	
Langer 2010	87.50%	26.56%	99.27%		Roux-en-Y bypass	
Mandeville 2017	57.14%	22.98%	85.63%		Roux-en-Y bypass	
Poghosyan 2016	87.50%	26.56%	99.27%		Roux-en-Y bypass	
Rheinwalt 2022	87.96%	80.37%	92.88%		Roux-en-Y gastric bypass or OAGB	
*Mortality*	*2.58%*	*1.36%*	*4.81%*	*0.00%*		
Felsenreich 2020	4.55%	0.28%	44.83%		Roux-en-Y bypass	
Bellorin 2021	2.38%	0.15%	28.74%		Roux-en-Y bypass	
Braghetto 2021	1.25%	0.08%	17.08%		Roux-en-Y bypass	
Carandina 2020	0.62%	0.04%	9.10%		Roux-en-Y bypass	
Chiappetta 2019	2.27%	0.14%	27.74%		Roux-en-Y bypass	
Curell 2021	1.39%	0.09%	18.67%		Roux-en-Y bypass	
D'Urso 2021	1.92%	0.12%	24.36%		Roux-en-Y bypass	
Huynh 2021	1.19%	0.07%	16.38%		Roux-en-Y bypass	
Iannelli 2016	4.17%	0.26%	42.46%		Roux-en-Y bypass	
Lim 2020	3.33%	0.21%	36.63%		Roux-en-Y bypass	
Parmar 2017	4.55%	0.28%	44.83%		Roux-en-Y bypass	
Termine 2021	3.12%	0.19%	35.0 3%		Roux-en-Y bypass	
Amiki 2020	6.25%	0.38%	53.86%		Roux-en-Y bypass	
Casillas 2016	1.52%	0.09%	20.08%		Roux-en-Y bypass	
Dijkhorst 2021	3.85%	0.24%	40.32%		Roux-en-Y bypass	
Landreneau 2018	2.78%	0.17%	32.21%		Roux-en-Y bypass	
Langer 2010	12.50%	0.73%	73.44%		Roux-en-Y bypass	
Poghosyan 2016	12.50%	0.73%	73.44%		Roux-en-Y bypass	
Rheinwalt 2022	0.46%	0.03%	6.90%		Roux-en-Y gastric bypass or OAGB	
*Complications (leak, DVT, blood loss, marginal ulcer, internal hernia, Endoscopic Mucosal injury, carcinoma *in situ*, or adenocarcinoma)*	*12.13%*	*3.95%*	*31.69%*	*0.00%*		
Bellorin 2021	15.38%	3.87%	45.06%		MSA	
Hawasli 2019	7.69%	1.07%	39.06%		MSA	
*Long-term symptom control (patient-reported;* > *6 months)*	*77.72%*	*36.37%*	*95.51%*	*68.74%*		
Bellorin 2021	92.31%	60.94%	98.93%		MSA	
Hawasli 2019	45.45%	20.28%	73.19%		MSA	
Broderick 2020	87.50%	46.27%	98.27%		MSA	
*Mortality*	*4.67%*	*1.17%*	*16.85%*	*0.00%*		
Bellorin 2021	3.57%	0.22%	38.39%		MSA	
Hawasli 2019	3.85%	0.24%	40.32%		MSA	
Broderick 2020	5.56%	0.34%	50.47%		MSA	
Desart 2015	6.25%	0.38%	53.86%		MSA	
*Reflux resolution baseline*	*45.45%*	*20.28%*	*73.19%*	*0.00%*		
Hawasli 2019	45.45%	20.28%	73.19%		MSA	
*Complications (leak, DVT, blood loss, marginal ulcer, internal hernia, Endoscopic Mucosal injury, carcinoma *in situ*, or adenocarcinoma)*	*15.11%*	*7.11%*	*29.29%*	*40.56%*		
Borbély 2018	2.78%	0.17%	32.21%		Other surgical or endoscopic interventions	Lower Esophageal sphincter electric stimulation
Debourdeau 2020	33.33%	8.39%	73.19%		Other surgical or endoscopic interventions	Anti-reflux mucosectomy technique using band ligation system
Hawasli 2021	4.55%	0.28%	44.83%		Other surgical or endoscopic interventions	Laparoscopic Ligamentum Teres cardiopexy
Khidir 2018	6.67%	0.93%	35.20%		Other surgical or endoscopic interventions	Stretta procedure
Macedo 2017	11.11%	1.54%	49.99%		Other surgical or endoscopic interventions	Hiatal hernia repair
Silecchia 2015	26.32%	11.40%	49.79%		Other surgical or endoscopic interventions	Fundectomy ± cruroplasty
Soong 2019	1.72%	0.11%	22.32%		Other surgical or endoscopic interventions	Hiatal hernia repair with Hill gastropexy
Walsh 2021	36.36%	14.33%	66.12%		Other surgical or endoscopic interventions	Endoscopic resection and plication technique
*Esophagitis present (Grade A, B, or C)*	*37.18%*	*2.24%*	*93.85%*	*86.54%*		
Debourdeau 2020	7.14%	0.43%	57.72%		Other surgical or endoscopic interventions	Anti-reflux mucosectomy technique using band ligation system
Soong 2019	93.75%	66.46%	99.13%		Other surgical or endoscopic interventions	Hiatal hernia repair with Hill gastropexy
Gálvez-Valdovinos 2015	13.33%	3.36%	40.54%		Other surgical or endoscopic interventions	Hiatal hernia repair with ligamentum teres cardiopexy buttress
*Long-term symptom control (patient reported;* > *6 months)*	*50.71%*	*29.26%*	*71.90%*	*74.14%*		
Borbély 2018	41.18%	21.04%	64.78%		Other surgical or endoscopic interventions	Lower Esophageal sphincter electric stimulation
Debourdeau 2020	50.00%	16.79%	83.21%		Other surgical or endoscopic interventions	Anti-reflux mucosectomy technique using band ligation system
Hawasli 2021	80.00%	45.93%	94.96%		Other surgical or endoscopic interventions	Laparoscopic Ligamentum Teres cardiopexy
Khidir 2018	20.00%	6.59%	46.98%		Other surgical or endoscopic interventions	Stretta procedure
Macedo 2017	22.22%	5.60%	57.90%		Other surgical or endoscopic interventions	Hiatal hernia repair
Silecchia 2015	97.50%	70.19%	99.85%		Other surgical or endoscopic interventions	Fundectomy ± cruroplasty
Soong 2019	25.00%	12.41%	43.95%		Other surgical or endoscopic interventions	Hiatal hernia repair with Hill gastropexy
Walsh 2021	9.09%	1.26%	43.86%		Other surgical or endoscopic interventions	Endoscopic resection and plication technique
Gálvez-Valdovinos 2015	86.67%	59.46%	96.64%		Other surgical or endoscopic interventions	Hiatal hernia repair with ligamentum teres cardiopexy buttress
Hawasli 2015	83.33%	36.87%	97.72%		Other surgical or endoscopic interventions	Anterior fundoplication, imbrication of fundus if dilated, re-sleeve if fundus and body of sleeve dilated
*Mortality*	*3.74%*	*1.56%*	*8.70%*	*0.00%*		
Borbély 2018	2.78%	0.17%	32.21%		Other surgical or endoscopic interventions	Lower Esophageal sphincter electric stimulation
Debourdeau 2020	7.14%	0.43%	57.72%		Other surgical or endoscopic interventions	Anti-reflux mucosectomy technique using band ligation system
Hawasli 2021	4.55%	0.28%	44.83%		Other surgical or endoscopic interventions	Laparoscopic Ligamentum Teres cardiopexy
Khidir 2018	3.12%	0.19%	35.03%		Other surgical or endoscopic interventions	Stretta procedure
Macedo 2017	5.00%	0.31%	47.49%		Other surgical or endoscopic interventions	Hiatal hernia repair
Silecchia 2015	2.50%	0.15%	29.81%		Other surgical or endoscopic interventions	Fundectomy ± cruroplasty
Soong 2019	1.72%	0.11%	22.32%		Other surgical or endoscopic interventions	Hiatal hernia repair with Hill gastropexy
Walsh 2021	4.17%	0.26%	42.46%		Other surgical or endoscopic interventions	Endoscopic resection and plication technique
Gálvez-Valdovinos 2015	3.12%	0.19%	35.03%		Other surgical or endoscopic interventions	Hiatal hernia repair with ligamentum teres cardiopexy buttress
Hawasli 2015	7.14%	0.43%	57.72%		Other surgical or endoscopic interventions	Anterior fundoplication, imbrication of fundus if dilated, re-sleeve if fundus and body of sleeve dilated

Italics indicate outcomes and cumulative event rates

### Acid exposure time (total)

There were two studies that evaluated medical treatment and one study that evaluated surgical treatment with bypass and its effect on acid exposure time. These were all single-arm studies. The mean time in the medical treatment group was 9.70 min (95% CI 6.15–13.25, *I*^2^ = 0.00%) and 3.80 min (95% CI 0.79–6.81 min, *I*^2^ = 0%).

### DeMeester core

There was only one single-arm medical study and two single-arm surgical bypass studies that evaluated the DeMeester score. In the medical arm, the mean score was 44.10 (95% CI 27.96–60.24, *I*^2^ = 0.00%), and in the surgical studies, the mean score was 30.5 (95% CI 21.35–59.69, *I*^2^ 90.38%).

### Impedance score post-intervention mean

Only a single study, Felsenreich et al. (2020) evaluated the impedance score in bypass surgical patients. The mean score was 16.30 (95% CI 13.62–18.98, *I*^2^ = 0%).

### Mean # of reflux episodes in 24 h

Only a single study, Felsenreich et al. (2020) evaluated the mean number of reflux episodes in 24 h in bypass surgical patients. The mean number of episodes was 49.00 (95% CI 26.13–71.87, *I*^2^ = 0%).

### Quality of life, GERD specific

There were six studies that evaluated GERD-specific quality of life in surgical patients, which included bypass, magnetic sphincter augmentation (MSA), or other surgical treatments, and 1 study that evaluated medical treatment specifically at 10 years. In Felsenreich et al. (2020), the Gastro-Intestinal Quality of Life Index was used, and the medical treatment had a mean score of 8.40 (95% CI 3.81–12.99, *I*^2^ = 0.00%). The mean scores in surgical patients varied among treatment modalities and in the used QoL scale. The three bypass studies had scores of 1.22 (95% CI 0.97–1.47, *I*^2^ = 0.00%, Visick Score) [24] 7.29 (95% CI 4.38–10.20, *I*^2^ = 0.00%) (GERD-health-related quality of life) [44], and 113.50 (95% CI 98.19–128.81, *I*^2^ = 0.00%) (Gastro-Intestinal Quality-of-Life Index) [38]. Two studies evaluated MSA, and two evaluated other procedures. In the MSA group, the mean score was 9.06 (95% CI 4.80–13.33, *I*^2^ = 0.00%) using the Health-Related Quality-of-Life score. The mean score in the other procedure group was 27.20 (95% CI − 1.30 to 55.70, *I*^2^ = 98.49%) using the GERD-health-related quality-of-life questionnaire. The large heterogeneity in the other treatment group is likely related to different procedures performed (Stretta versus endoscopic interventions with or without plication).

### Quality of life, GERD specific at 1, 6, and 12 months

Soong et al. evaluated QoL at 1, 6, and 12 months after hiatal hernia repair with gastropexy using the GERD-health-related quality-of-life questionnaire and found mean scores of 12.30 (95% CI 7.83–16.77, *I*^2^ = 0%), 16.80 (95% CI 10.47–23.13, *I*^2^ = 0%), and 17.40 (95% CI 9.56–25.24, *I*^2^ = 0%), respectively.

### Quality of life, overall

Three studies evaluated post-surgical patients, one each of bypass, MSA or Stretta, and their QoL overall. Again, comparison between these three studies is difficult as the same scale was not used. The bypass group used the Gastro-Intestinal Quality-of-Life Index with a mean score of 5.10 (95% CI 3.64–6.56, *I*^2^ = 0.00%), the MSA group on the GERD score questionnaire had a mean score of 5.14 (95% CI 4.86–5.42, *I*^2^ = 0.00%), and the Stretta group on the GERD-health-related quality-of-life scale had a mean score of 41.80 (95% CI 36.23–47.37, *I*^2^ = 0.00%).

### Barrett’s esophagus

Three studies evaluated regression of Barrett’s esophagus in patients treated medically, and one study assessed this outcome after surgery. The medically treated patients had an event rate of 8.42% (95% CI 3.41–19.35%, *I*^2^ = 54.87%) which was lower than the single study of patients treated with bypass (20.00%, 95% CI 5.04–54.07%, *I*^2^ = 0.00%).

### Complications (leak, DVT, blood loss, marginal ulcer, internal hernia, endoscopic mucosal injury, carcinoma in situ, or adenocarcinoma)

**Fig. 5:**
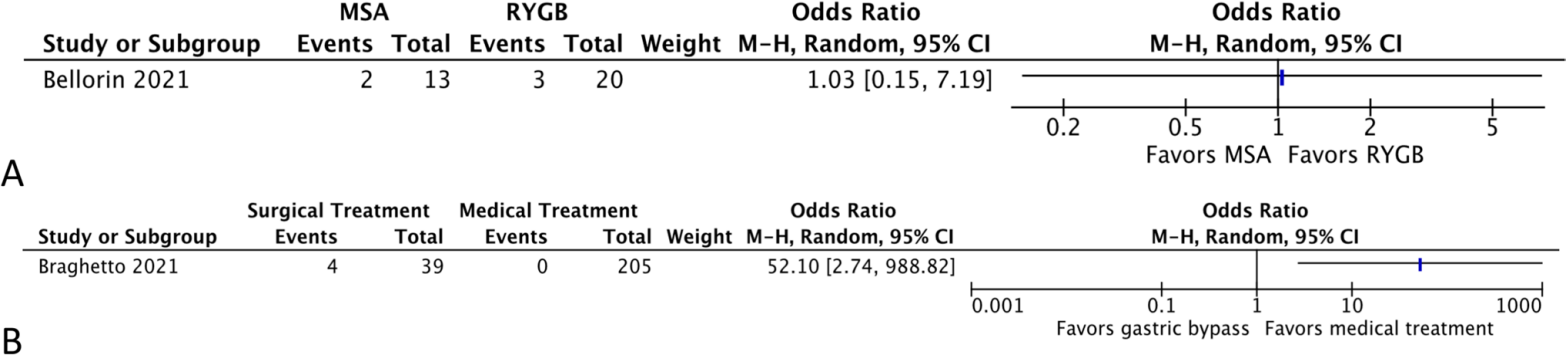
Forest plots evaluating comparisons of complication rates in MSA versus RYGB (**A**) and RYGB versus medical treatment (**B**)

A single study evaluated patients treated medically and their rate of complications (0.24%, 95% CI 0.02–3.76%, *I*^2^ = 0.00%). In surgical patients that were stratified by type of surgery performed, bypass patients had the highest complication rate (15.21%, 95% CI = 11.73–19.49%, *I*^2^ = 6.88%, 13 studies), compared to MSA (12.13%, 95% CI 3.95–31.69%, *I*^2^ = 0.00%) and other procedures (12.13%, 95% CI 3.95–31.69%, *I*^2^ = 0.00%). The odds ratio was non-significant between MSA and bypass (1.03, 95% CI 0.15–7.19) with a propensity for bypass (Fig. 5A). When comparing medical treatment to bypass, the odds ratio was 52.10 (95% CI 2.74–988.82), favoring medical treatment (Fig. 5B).

### Esophagitis present (Grade A, B, or C)

There were three studies of medical treatment that evaluated esophagitis compared to 12 studies that evaluated surgical treatment and postoperative esophagitis. The event rate of the medically treated patients was 29.02% (95% CI 21.48–37.92%, *I*^2^ = 17.02%) compared to 15.00% with bypass (95% CI 6.42–31.21%, *I*^2^ = 62.41%) and 37.18% with other surgical/endoscopic treatments (95% CI 2.24–93.85%, *I*^2^ = 86.54%).

### Long-term symptom control (patient = reported; > 6 months)

**Fig. 6:**
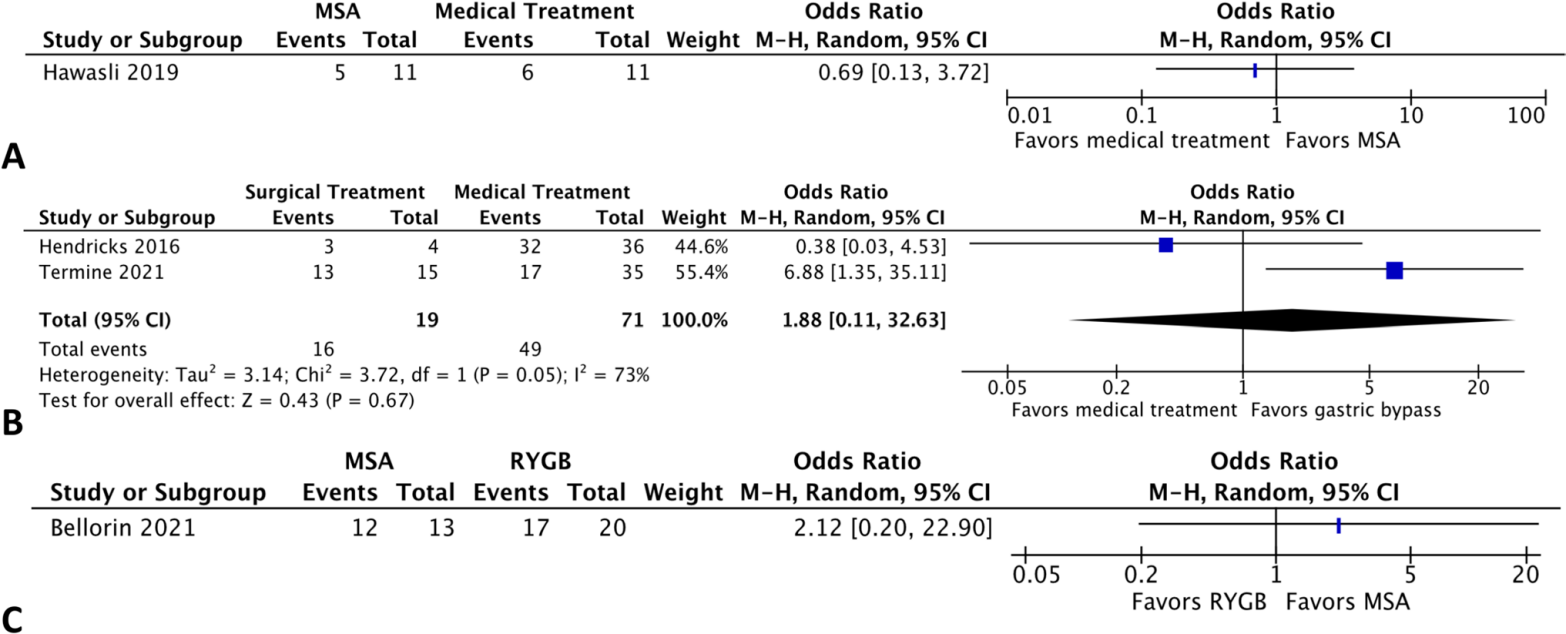
Comparative studies evaluating MSA versus medical treatment (**A**), medical treatment versus RYGB (**B**), and MSA versus RYGB (**C**)

There were four studies that evaluated patients treated medically compared to 33 studies that compared postoperative surgical interventions. In the medically treated group, the rate of long-term symptom control was 71.54% (95% CI 48.96–86.82%, *I*^2^ = 85.83%). In surgical patients, the event rate varied from 50.71% in the other treatment group (95% CI 29.26–71.90%, *I*^2^ = 74.14%) to 77.72% with magnetic sphincter augmentation (95% CI = 36.37–95.51%, *I*^2^ = 68.74%) to 80.05% with bypass (95% CI 73.77–85.13%, *I*^2^ = 37.17%). Comparing medical treatment to MSA, the odds ratio was 0.69 (95% CI 0.13–3.72, Fig. 6A), favoring medical treatment, while bypass, compared to medical treatment, had an odds ratio of 1.88 (95% CI 0.11–32.63, Fig. 6B), favoring bypass, neither of which were significant. When comparing the MSA to RYGB, the OR was 2.12 (95% CI 0.20–22.90, Fig. 6C), favoring MSA.

### Mortality

No studies evaluated mortality in the medically treated group. Multiple studies commented on mortality in each surgically stratified group. In the other treatments group, the event rate was 3.74% (95% CI 1.56–8.70%, *I*^2^ = 0.00%), 4.67% in the magnetic sphincter augmentation group (95% CI 1.17–16.85%, I^2^ = 0.00%) and 2.58% in the bypass group (95% CI 1.36–4.81%, *I*^2^ = 0.00%).

### Question 3) Should SG versus intestinal bypass procedures be used in patients with obesity with inflammatory bowel disease (IBD)?

**Fig. 7:**
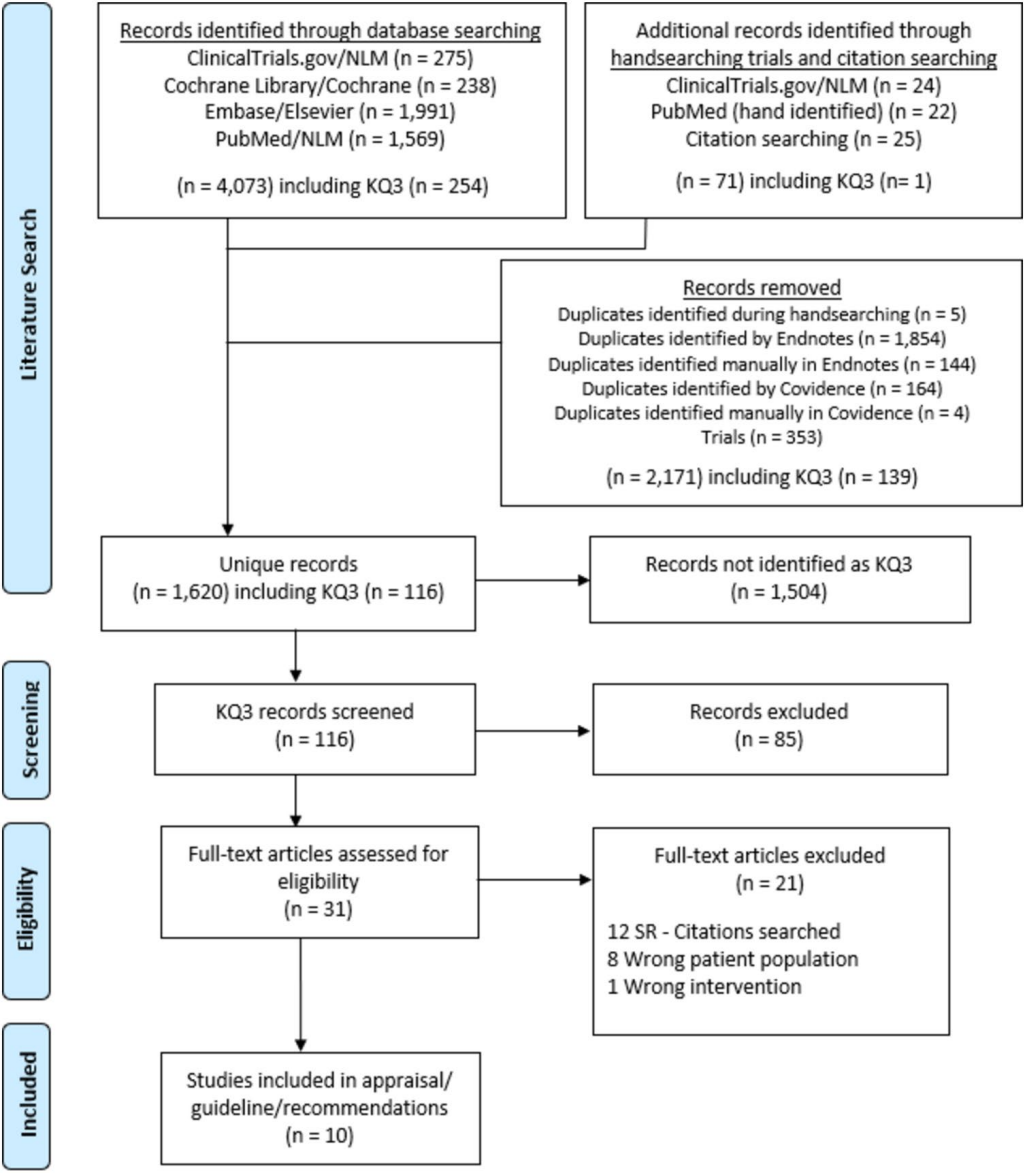
PRISMA diagram for KQ3

**Fig. 8:**
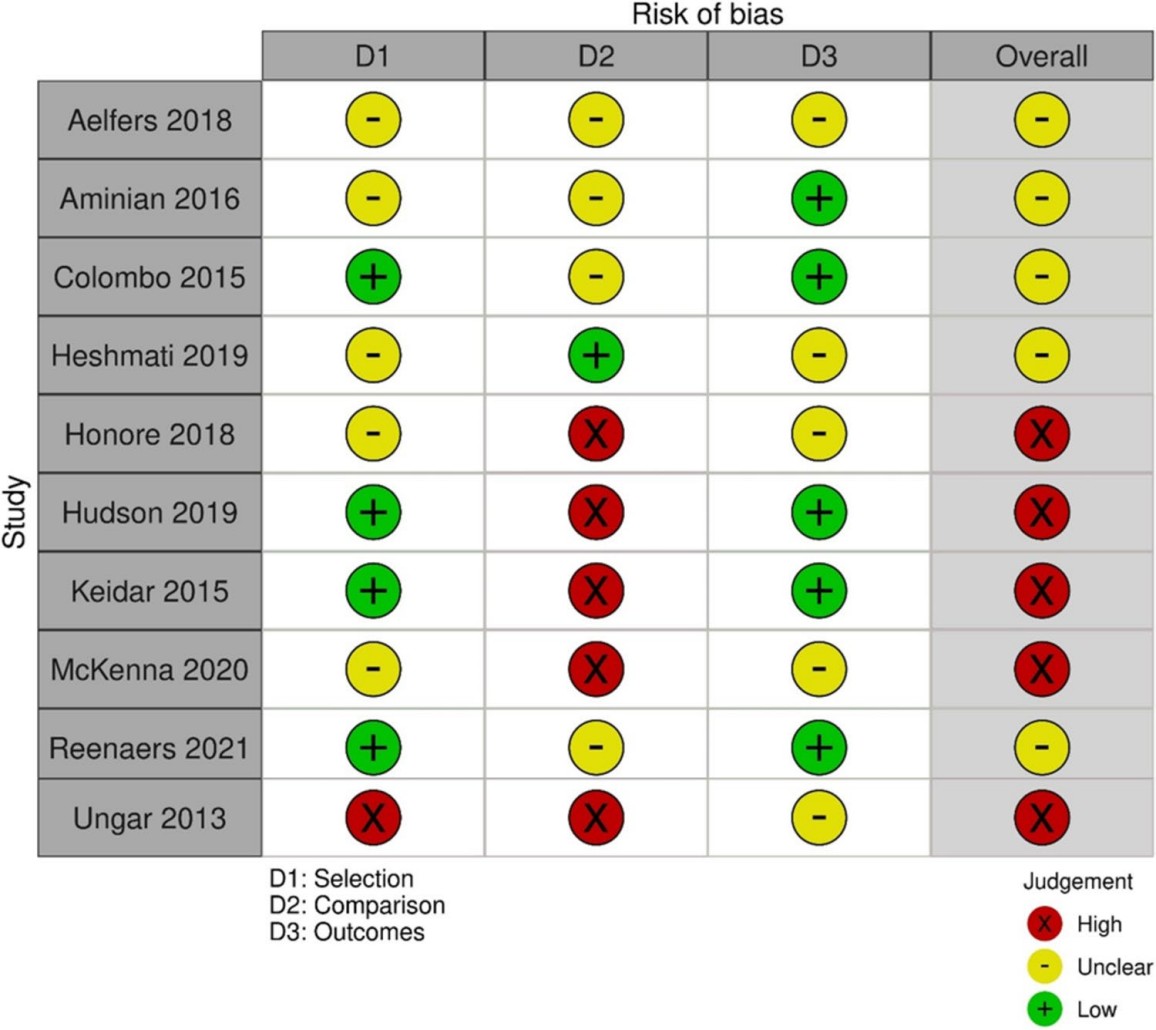
Stoplight diagram for KQ3 studies demonstrating the risk of bias

A literature search was completed for KQ3 which yielded 116 articles after removal of duplications (*n* = 5). After title and abstract screening that resulted in 31 articles for full-text screening and then 10 articles for data extraction. Ten articles were included in the final meta-analysis (Table 9) [60–69]. The PRISMA flow diagram for the systematic review is seen in Fig. 7. The risk of bias assessment of each study is found in Fig. 8. The results are found in Tables 10 and 11. In addition to the reported outcomes, we also attempted to extract data related to IBD quality of life, and drug serious adverse effects but found no data related to the aforementioned outcomes.

**Table 9:** Studies included in KQ3

First author last name	Year of publication	Funding source	Study design	Number of Crohn’s Patients	Number of UC patients	Number of sleeve	Number of bypass
Ungar [60]	2013	None specified	Single-center retrospective cohort	4	0	4	0
Reenaers [61]	2021	None specified	Multicenter retrospective cohort	66	22	73	3
McKenna [62]	2020	None specified	Multicenter retrospective cohort	11	20	14	14
Keidar [63]	2015	None specified	Retrospective cohort	8	2	9	0
Hudson [64]	2019	Crohn’s and Colitis foundation	Single-center retrospective cohort	8	4	9	3
Honoré [65]	2018	None specified	Multicenter retrospective cohort	8	0	8	0
Heshmati [66]	2019	None specified	Single-center retrospective cohort	31	23	35	19
Colombo [67]	2015	None specified	Single-center retrospective cohort	4	1	5	0
Aminian [68]	2016	None specified	Single-center retrospective cohort	9	8	7	13
Aelfers [69]	2018	None specified	Single-center retrospective cohort	27	15	23	9

**Table 10:** Sleeve gastrectomy results in patients with IBD

Study name	Event rate	Lower limit	Upper limit	*I*^2^	*N*
*IBD-associated complications*	*5.89%*	*2.22%*	*14.68%*	*0.00%*	
Aminian 2016	5.00%	0.31%	47.49%		9
Colombo 2015	8.33%	0.50%	62.18%		5
Heshmati 2019	5.71%	1.43%	20.16%		35
McKenna 2020	3.33%	0.21%	36.63%		14
Ungar 2013	10.00%	0.59%	67.36%		4
*Pain requiring medical therapy*	*6.05%*	*2.41%*	*14.36%*	*0.00%*	
Aminian 2016	5.00%	0.31%	47.49%		9
Colombo 2015	8.33%	0.50%	62.18%		5
Heshmati 2019	2.86%	0.40%	17.69%		35
Ungar 2013	10.00%	0.59%	67.36%		4
Honoré 2018	5.56%	0.34%	50.47%		8
Hudson 2019	5.00%	0.31%	47.49%		9
Keidar 2015	11.11%	1.54%	49.99%		
*Subjective recurrence of IBD*	*7.23%*	*2.89%*	*16.96%*	*0.00%*	
Heshmati 2019	2.86%	0.40%	17.69%		35
Ungar 2013	10.00%	0.59%	67.36%		4
Keidar 2015	11.11%	1.54%	49.99%		9
Aelfers 2018	8.70%	2.18%	28.88%		23
*Long-term complications (dumping syndrome, malabsorption, leaks, fistulas, *etc.*)*	*5.32%*	*2.61%*	*10.51%*	*0.00%*	
Aminian 2016	5.00%	0.31%	47.49%		9
Colombo 2015	8.33%	0.50%	62.18%		5
Heshmati 2019	5.71%	1.43%	20.16%		35
Ungar 2013	10.00%	0.59%	67.36%		4
Honoré 2018	5.56%	0.34%	50.47%		8
Hudson 2019	5.00%	0.31%	47.49%		9
Keidar 2015	11.11%	1.54%	49.99%		9
Reenaers 2021	2.74%	0.69%	10.30%		73
*Mortality (all cause)*	*4.35%*	*1.96%*	*9.40%*	*0.00%*	
Aminian 2016	11.11%	1.54%	49.99%		9
Colombo 2015	8.33%	0.50%	62.18%		5
Heshmati 2019	2.86%	0.40%	17.69%		35
McKenna 2020	3.33%	0.21%	36.63%		14
Ungar 2013	10.00%	0.59%	67.36%		4
Honoré 2018	5.56%	0.34%	50.47%		8
Hudson 2019	5.00%	0.31%	47.49%		9
Keidar 2015	5.00%	0.31%	47.49%		9
Aelfers 2018	2.08%	0.13%	25.94%		23
Reenaers 2021	0.68%	0.04%	9.89%		73
*Perioperative complications (*< *30d) Clavien dindo* = *2*	*11.09%*	*6.47%*	*18.36%*	*8.63%*	
Aminian 2016	33.33%	11.11%	66.66%		9
Colombo 2015	20.00%	2.72%	69.10%		5
Heshmati 2019	2.86%	0.40%	17.69%		35
Ungar 2013	25.00%	3.35%	76.22%		4
Honoré 2018	5.56%	0.34%	50.47%		8
Hudson 2019	5.00%	0.31%	47.49%		9
Keidar 2015	11.11%	1.54%	49.99%		9
Aelfers 2018	4.35%	0.61%	25.22%		23
Reenaers 2021	9.59%	4.64%	18.78%		73
*Reoperations related to bariatric procedure*	*5.35%*	*2.74%*	*10.22%*	*0.00%*	
Aminian 2016	5.00%	0.31%	47.49%		9
Colombo 2015	8.33%	0.50%	62.18%		5
Heshmati 2019	1.39%	0.09%	18.67%		35
McKenna 2020	3.33%	0.21%	36.63%		14
Honoré 2018	5.56%	0.34%	50.47%		8
Hudson 2019	5.00%	0.31%	47.49%		9
Keidar 2015	11.11%	1.54%	49.99%		9
Aelfers 2018	2.08%	0.13%	25.94%		23
Reenaers 2021	4.11%	1.33%	11.98%		73
Ungar 2013	25.00%	3.35%	76.22%		4

Italics indicate outcomes and cumulative event rates

**Table 11:** Outcomes in patients treated with bypass with concurrent IBD

Study name	Event rate	Lower limit	Upper limit	*I*^2^	*N*
*IBD-associated complications*	*11.24%*	*4.24%*	*26.58%*	*0.00%*	
Aminian 2016	5.56%	0.34%	50.47%		8
Heshmati 2019	15.79%	5.18%	39.15%		19
McKenna 2020	3.33%	0.21%	36.63%		14
*Pain requiring medical therapy*	*17.30%*	*7.34%*	*35.60%*	*0.00%*	
Aminian 2016	5.56%	0.34%	50.47%		8
Heshmati 2019	21.05%	8.13%	44.55%		19
Hudson 2019	12.50%	0.73%	73.44%		3
*Subjective recurrence of IBD*	*20.57%*	*2.71%*	*70.65%*	*59.71%*	
Heshmati 2019	36.84%	18.68%	59.70%		19
Aelfers 2018	5.00%	0.31%	47.49%		9
*Long-term complications (dumping syndrome, malabsorption, leaks, fistulas, *etc.*)*	*18.25%*	*9.55%*	*32.08%*	*0.00%*	
Aminian 2016	25.00%	6.30%	62.29%		8
Heshmati 2019	15.79%	5.18%	39.15%		19
McKenna 2020	14.29%	3.60%	42.68%		14
Hudson 2019	33.33%	4.34%	84.65%		3
Reenaers 2021	12.50%	0.73%	73.44%		3
*Mortality (all cause)*	*5.67%*	*1.83%*	*16.26%*	*0.00%*	
Aminian 2016	5.56%	0.34%	50 .47%		8
Heshmati 2019	2.50%	0.15%	29.81%		19
McKenna 2020	3.33%	0.21%	36.63%		14
Hudson 2019	12.50%	0.73%	73.44%		3
Aelfers 2018	5.00%	0.31%	47.49%		9
Reenaers 2021	12.50%	0.73%	73.44%		3
*Perioperative complications (*< *30d) Clavien dindo* = *2*	*26.83%*	*13.91%*	*45.41%*	*12.10%*	
Aminian 2016	50.00%	20.01%	79.99%		8
Heshmati 2019	10.53%	2.65%	33.74%		19
Hudson 2019	33.33%	4.34%	84.65%		3
Aelfers 2018	22.22%	5.60%	57.90%		9
Reenaers 2021	33.33%	4.34%	84.65%		3
*Reoperations related to bariatric procedure*	*16.99%*	*9.01%*	*29.73%*	*0.00%*	
Aminian 2016	5.00%	0.31%	47.49%		8
Heshmati 2019	10.53%	2.65%	33.74%		19
McKenna 2020	21.43%	7.07%	49.43%		14
Hudson 2019	12.50%	0.73%	73.44%		3
Aelfers 2018	22.22%	5.60%	57.90%		9
Reenaers 2021	33.33%	4.34%	84.65%		3

Italics indicate outcomes and cumulative event rates

### IBD-associated complications

**Fig. 9:**
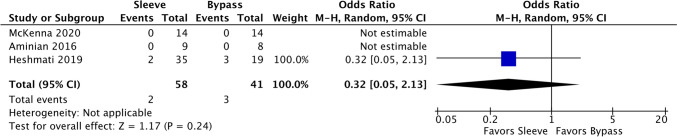
Forest plot demonstrating the comparative studies demonstrating non-significant favoring of sleeve gastrectomy

There were 3 comparative studies and 2 single-arm studies of sleeve patients that evaluated worsening of IBD specifically looking at refractory disease or complications associated with the disease, including obstruction, hemorrhage, fistula, or perforation. Sleeve had an event rate of 5.89% (CI 2.22–14.68%, *I*^2^ = 0.00%) whereas bypass had an event rate of 11.24% (CI 4.24–26.58%, *I*^2^ = 0.00%). Only Heshmati et al. of the comparative studies was used to estimate the odds ratio (OR 0.32 in favor of sleeve 95% CI 0.05–2.13, Fig. 9), as the other two studies had no occurrences of the outcome of interest.

### Pain requiring medical therapy

**Fig. 10:**
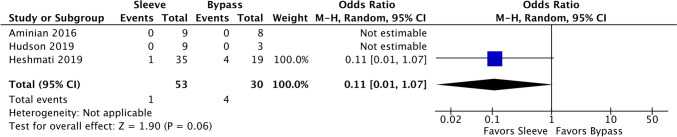
Forest plot of comparative studies for IBD worsening requiring medical treatment of pain

There were three comparative studies and four sleeve-only single-arm studies that evaluated worsening of pain requiring medical therapy. Sleeve had an event rate of 6.05% (95% CI 2.41–14.36% *I*^2^ = 0.00%) which was lower than bypass at 17.30% (CI 7.34–35.60%, *I*^2^ = 0.00%) but did not achieve statistical significance. When evaluating the comparative studies, only Heshmati et al. reported any events, with an OR of 0.11 in favor of sleeve (95% CI 0.01–1.07, Fig. 10).

### Subjective recurrence of IBD

**Fig. 11:**
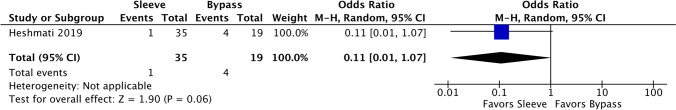
Forest plot of comparative studies for IBD worsening that is patient reported

Two comparative studies and two single-arm studies evaluated sleeve patients. The event rate trended towards being lower in the sleeve patients (7.23%, CI = 2.89–16.96%, *I*^2^ = 0.00%) compared to the bypass patients (20.57%, CI 2.71–70.65%, *I*^2^ = 59.71%). Of the comparative studies, only Heshmati et al. reported events in both groups accounting for 100% of the weight of the odds ratio of 0.11 in favor of sleeve (95% CI 0.01–1.07, Fig. 11).

### Long-term complications (dumping syndrome, malabsorption, leaks, fistulas, etc.)

**Fig. 12:**
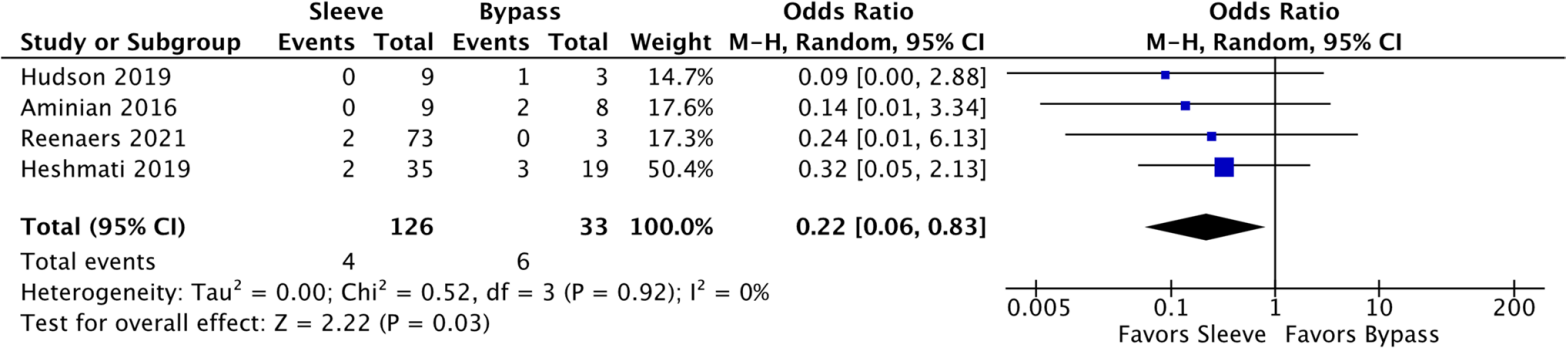
Forest plot of comparative studies of long-term complications

There were four comparative studies, four single-arm studies on sleeve patients, and one single-arm study on bypass patients. The event rate of the sleeve patients (5.32%, 95% CI 2.61–10.51%, *I*^2^ = 0.00%) was lower than that of the bypass patients (18.25%, 95% CI 9.55–32.08%, *I*^2^ = 0.00%), and sleeve gastrectomy was favored (Fig. 12). Among the comparative studies, the OR was 0.22 (95% CI 0.06–0.83, *I*^2^ = 0%), indicating a lower odd of complications with sleeve gastrectomy.

### Mortality (all-cause)

**Fig. 13:**
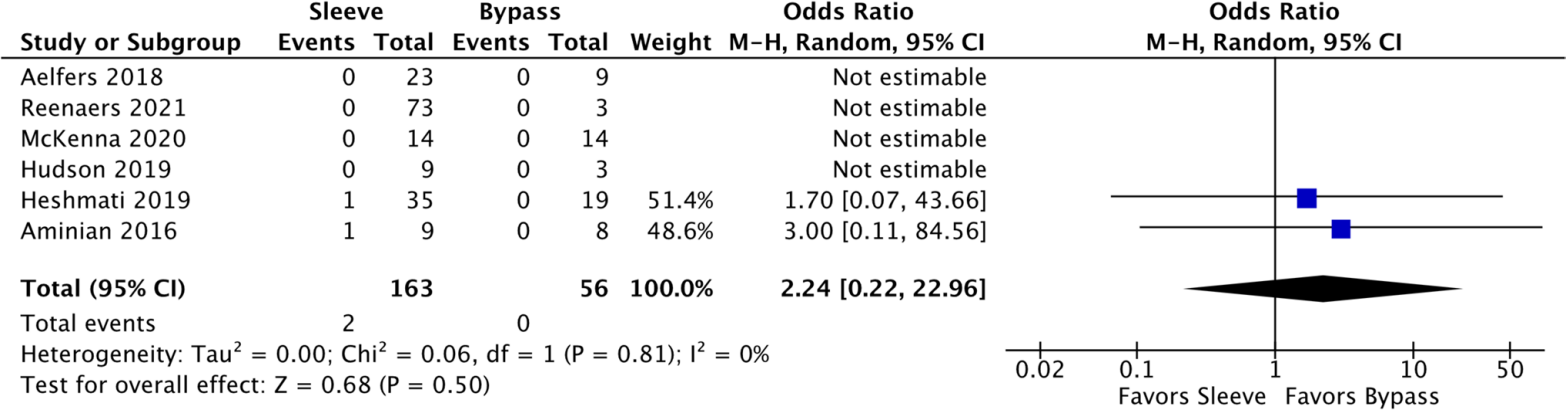
Forest plot of comparative studies that evaluated mortality

There were six comparative studies and four single-arm sleeve studies. Of the six comparative studies, only Heshmati et al. and Arminian et al. were included in the odds ratio calculation as the remaining four had no events. This was the only outcome that favored bypass (OR 2.24, 95% CI 0.22–22.96, Fig. 13). However, it was also non-significant and had a wide confidence interval. However, the overall events rate for sleeve patients was lower at 4.35% (95% CI 1.96–9.40%, *I*^2^ = 0.00%) compared to bypass at 5.67% (1.83–16.26%, *I*^2^ = 0.00%).

### Perioperative complications (< 30d) Clavien dindo ≥ 2

**Fig. 14:**
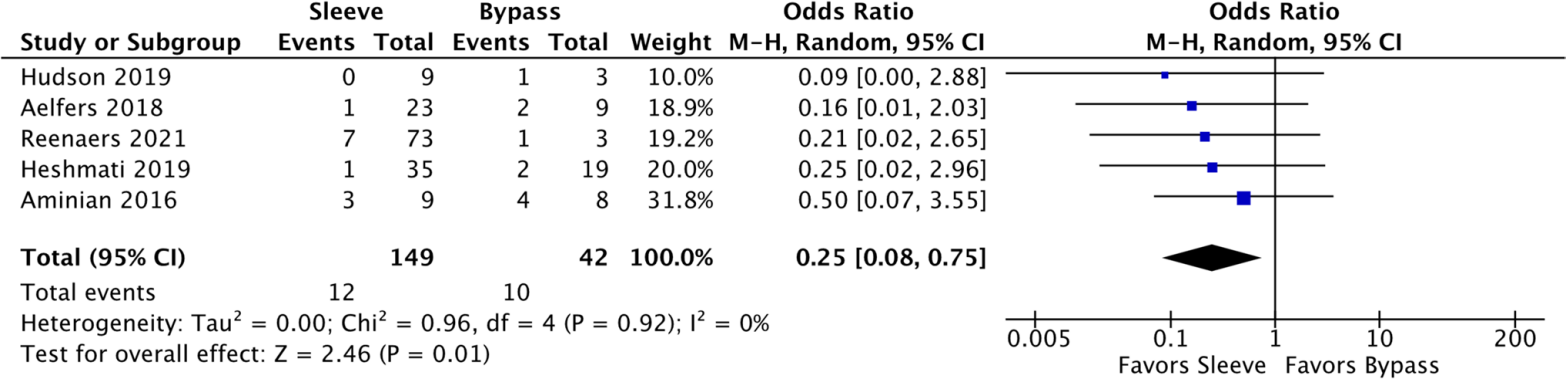
Forest plot of comparative studies evaluating perioperative complications

There were five comparative studies and four single-arm studies of sleeve patients. Sleeve gastrectomies had fewer perioperative complications (OR 0.25, 95% CI 0.08–0.75, Fig. 14). The overall event rate in sleeves was 11.09% (95% CI 6.47–18.36%, *I*^2^ = 8.63%) and in bypass was 26.83% (95% CI 13.91–45.41%, *I*^2^ = 12.10%).

### Reoperations related to bariatric procedure

**Fig. 15:**
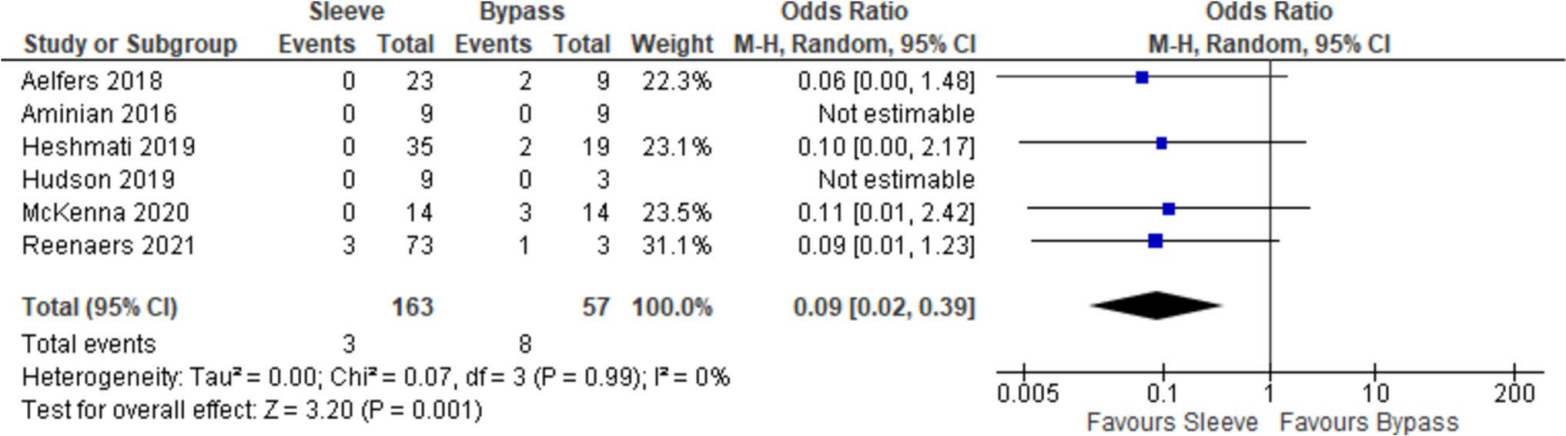
Forest plot of the comparative studies evaluating reoperation rates

There were six comparative studies that evaluated reoperation in the setting of failure of primary bariatric procedure, IBD surgery or both, and three single-arm studies evaluating sleeve patients for reoperations. Four of the comparative studies were used for the odds ratio calculation, which favored sleeve which was statistically significant (OR 0.09, 95% CI 0.02–0.39, Fig. 15). The overall event rate for sleeve patients was 7.00% (95% CI 4.01–11.95%, *I*^2^ = 0.00%) and 14.91% in bypass patients (95% CI 7.57–27.27%, *I*^2^ = 0.00%). Reoperation indications included internal hernias (*n* = 4), sleeve leaks (*n* = 2), gastric stricture (*n* = 1), mesenteric ischemia/small bowel perforation (*n* = 1), biliopancreatic limb obstruction (*n* = 1), bile reflux (*n* = 1), and gastro-gastric fistula (*n* = 1).

## Discussion

### Gallstones and bariatric surgery

Common bile duct stones in a patient with bypass-type anatomy are a challenging problem. Due to the altered anatomy, standard ERCP cannot be performed. In these cases, other potential options include double-balloon enteroscopy, operative gastrotomy with ERCP, or endoscopic ultrasound-directed transgastric ERCP, all of which are associated with additional morbidity. We sought to evaluate if the routine use of IOC at the time of cholecystectomy was beneficial. No randomized controlled trials and few comparative observational studies have addressed this question.

Routine use of IOC was associated with a significantly decreased rate of CBD injury and a trend towards decreased intraoperative complications, perioperative complications, and mortality, although not statistically significant. There was also a trend towards lower use of ERCP intraoperatively in the routine IOC group. Unfortunately, no studies evaluated the use of ERCP postoperatively in the non-routine use of IOC patients. The use of ERCP intraoperatively was higher in the group undergoing sleeve gastrectomies than bypass; however, when evaluating the ERCP rate postoperatively, this was higher in the patients who had undergone bypass. The rate of reoperation, postoperative pancreatitis, cholangitis, and choledocholithiasis was low in the routine use of the IOC group but no non-routine IOC studies evaluated these outcomes, so it is impossible to say whether these outcomes were decreased.

### GERD post-sleeve gastrectomy

Much has been written about the persistence or worsening of reflux symptoms after sleeve gastrectomy. There are multiple management options, ranging from medical therapy with PPIs to endoscopic and surgical interventions. We sought to determine if medical or surgical management was superior. Objective measures of GERD after sleeve gastrectomy, including acid exposure time and DeMeester Score, favored surgical treatment, although neither outcome was statistically significant. Other objective measures were only available for surgical outcomes, including impedance score and mean number of reflux episodes, making their value challenging to interpret. Alternatively, medical treatment resulted in lower rates of Barrett’s esophagus, though this was limited by a paucity of data on surgical patients. Regarding patient-reported outcomes, GERD-specific quality of life was significantly higher in the surgically treated group. In Soong et al., quality of life increased with time after surgery. The long-term patient-reported symptom control showed medical treatment inferiority to bypass, similarity to MSA, and superiority to other surgical/endoscopic treatments (see Table 8); however, none reached significance. Definitive conclusions are challenging based on the limited data. While many patients may benefit from conversion to gastric bypass for post-sleeve gastrectomy GERD, several factors must be considered prior to this decision. These include and are not limited to suboptimal clinical response, the presence of a hiatal hernia, abnormal GI motility, and psychosocial issues that may preclude conversion to RYGB. The complexity of this decision underlines the importance of multidisciplinary evaluation prior to pursuing a bypass-type operation.

### IBD and bariatric surgery

As the rates of both obesity and IBD increase [1, 70], determining the optimal surgical weight loss procedure in patients with both diseases is imperative. We sought to determine if there was a bariatric procedure that had improved outcomes in IBD patients. In our review, surgical bypass had worse outcomes measured in terms of IBD sequelae compared to sleeve gastrectomy including obstruction, hemorrhage, fistula, perforation, pain, patient perception, and ulceration. Patients with IBD who underwent sleeve gastrectomy also had lower complication rates both long and short term. Mortality was the only outcome that favored bypass in these patients; however, this was a rare event, making this effect estimate very fragile. The reoperation rate was higher in bypass patients. While none of the direct comparative studies had statistical significance, the overall data are compelling evidence that a sleeve gastrectomy should be the first surgical option offered to these patients.

A similar meta-analysis has evaluated IBD and bariatric surgery and found that it is safe in these patients [71]. Interestingly, in that study, they also evaluated the timing of the bariatric procedure in relation to the diagnosis of IBD and found that bariatric surgery may be a risk factor for developing IBD [71].

## Limitations

This review had several limitations. Overall, the lack of randomized controlled trials and even direct, comparative studies makes drawing accurate conclusions difficult. There were several outcomes for each question that were missing data from an individual arm where conclusions were impossible.

There were also several outcomes with significant heterogeneity noted in the pooled data. In general, the study quality for these questions was poor, limiting the generalizability of this data. Much of the data analyzed in this review has been retrospectively collected or prospectively collected into databases that are searched retrospectively. Many of the studies gathered were determined to be at high risk of bias, which is a known problem with observational studies.

There was significant variability in the outcomes that study authors chose to report.

Specifically relating to the question of IBD, a perfectly designed question would distinguish the UC and CD populations, given their different pathophysiology and complication profile. However, most publications on the subject lump the patient populations together since there is such a small sample size. Given the limitations of the data, we did include all patients with IBD. Even doing so, we had very limited data to work with. In the design of the questions, we did not have a colorectal surgery representative included in formulating how we should evaluate the IBD patients which could have lead to optimized outcomes assessment.

## Future research recommendations

There is much room for improvement in research regarding obesity-associated medical conditions in the setting of metabolic and bariatric surgical procedures. Many of the studies included in this review are single-arm retrospective reviews which provide the most biased outcomes for any of the questions posed. High-quality, randomized trials are needed to evaluate the best treatment and management methods for these comorbidities including routine use of IOC in patients who are undergoing or have undergone bariatric surgery, optimal surgical management of IBD, and treatment of GERD after sleeve gastrectomy.

As research collaborations are established, the gathering of high-quality data on a large number of patients would be beneficial. In addition, evaluating patient databases that are not solely for bariatric surgery but also for comorbid conditions such as IBD can be utilized to broaden the patients available for analysis. It is also essential to report outcomes for UC and CD patients independently of each other in all studies on the intersection of IBD and metabolic and bariatric surgery.

## Conclusion

Our review summarizes three important questions regarding comorbid conditions in the setting of bariatric surgery. The routine use of intraoperative cholangiogram (IOC) in patients undergoing bypass-type bariatric surgery or who have undergone bariatric surgery and are now undergoing cholecystectomy is supported by the limited data presented. In addition, treatment of GERD after sleeve gastrectomy favored surgical treatment but this depended on the surgical treatment used, and thus, further stratification is needed. Finally, in patients with IBD, the procedure associated with the fewest complications is sleeve gastrectomy. Given the overwhelming number of single-arm studies that were used in this pooled data, there is a need for more direct comparative studies and high-quality randomized controlled trials for more generalizable data. The findings from this review will inform the SAGES Guidelines on the management of comorbidities for bariatric surgery patients.

## Disclosures

Bradley Kushner, Theofano Zoumpou, Julietta Chang, Adam Reid, L Renee Hilton, Omar Ghanem, Andrew Sabour, Lindsay Loss, Essa Aleassa, Noe Rodriguez, Subhashini Ayloo, Sunjay Kumar, D. Wayne Overby, and Claire Wunker have no disclosures. Peter Hallowell is President-Elect Midwest Surgical Association and on the Board of directors for Piedmont Liability Trust. Ivy N. Haskins receives royalties from Up To Date, Inc (unrelated to this work). Tammy Kindel has grants with the NHLBI and American College of Surgeons, receives honoraria from Translational Medicine Academy, Medtronic, and Intuitive, has patents pending under U.S. Provisional Patent Application No. 63/505,036 and does unpaid work for Treo Foundation. Varun Bansal has a grant from Yale University (unrelated to this work).

## Supplementary Information

Below is the link to the electronic supplementary material.

Supplementary file 1 (DOCX 30 kb)
