# Supplementary material for: Bariatric surgery and relevant comorbidities: a systematic review and meta-analysis
Source: Surg Endosc. 2025 Feb 7;39(3):1419–48. doi: 10.1007/s00464-025-11528-4 (PMC11870965; doi:10.1007/s00464-025-11528-4)
Supplement: Supplementary file 1 — (DOCX 30 kb) [file 464_2025_11528_MOESM1_ESM.docx]

# Guidelines for the Management of Bariatric Surgeries

# Literature Searches Summary Chart

(Updated: 7/27/23)

## KQ1

| 1. **Should routine IOC versus alternative options be used for patients undergoing cholecystectomy with current or previous gastrointestinal bypass type bariatric procedures (RYGB and DS, etc.)?** | | |
| --- | --- | --- |
| **Database** | **Final search strategies** |  |
| PubMed  Q1 | ((cholecystectomy [mh] OR cholecystectomy [tw] OR cholecystectomies [tw]) AND (Bariatric Surgery [mh] OR "bariatric surgery" [tw] OR gastric bypass[tw] OR Gastroenterostomy[mh] OR Roux-en-Y[tw] OR "Anastomosis, Roux-en-Y"[mh] OR OAGB[tw] OR "anastomosis gastric"[tw] OR duodenum/surgery[mh] OR duodenal switch[tw] OR SADI[tw] OR "single anastomosis"[tw] OR Gastroenterostomy[mh]) AND (Clinical Study[pt] OR Comparative Study[pt] OR Epidemiologic Studies[mh] OR Evaluation Study[pt] OR Meta-Analysis[pt] OR Multicenter Study[pt] OR Systematic Review[pt] OR randomized[tiab] OR study[tiab] OR studies [tiab] OR follow-up studies[mh] OR Practice Guideline[pt] OR "practice guidelines as topic"[mh] OR guideline[title] OR guidelines[title])) NOT (Case reports[pt] OR "case report"[tw] OR ("animals"[MH:noexp] NOT "humans"[MH]) OR rat[tiab] OR rats[tiab] OR dog[tiab] OR dogs[tiab] OR porcine[tiab] OR "infant"[MeSH] OR (child[mh] NOT adult[mh]) OR children[title] OR child[title] OR infants[title] OR infant[title] OR pediatric [tiab] OR (1889:1989 [ppdat])) |  |
| Embase  Q1 | (('cholecystectomy'/exp OR cholecystectomy) AND ('bariatric surgery'/exp OR 'bariatric surgery' OR 'roux en y' OR 'roux y anastomosis'/exp OR 'roux y anastomosis' OR 'roux-en-y gastric bypass'/exp OR 'roux-en-y gastric bypass' OR 'biliopancreatic bypass'/exp OR 'biliopancreatic bypass' OR 'duodenal switch' OR 'single anastomosis gastric bypass'/exp OR 'single anastomosis gastric bypass') AND ([adult]/lim OR [aged]/lim OR [middle aged]/lim OR [very elderly]/lim OR [young adult]/lim) AND ('practice guideline'/exp OR 'practice guideline' OR [cochrane review]/lim OR [systematic review]/lim OR [meta analysis]/lim OR [clinical study]/lim)) NOT ([conference abstract]/lim OR 'case report'/exp OR 'case report' OR [animals]/lim OR [1889-1989]/py) |  |
| Cochrane  Q1 | cholecystectomy AND (bariatric surgery OR Gastric bypass OR Roux-en-y OR SADI OR OAGB) |  |
| Clinical Trials Q1 | cholecystectomy AND (bariatric surgery OR gastric bypass OR SADI OR OAGB OR duodenum OR roux-en-y) |  |
| PubMed Run 2 | Above NOT ("1960/01/01"[CRDT] : "2020/07/20"[CRDT]) |  |
| Embase Run 2 | Above NOT [1889-2019]/py |  |
| Cochrane Run 2 | Above with Cochrane Library publication date from Jul 2020 to Feb 2022 |  |
| Clinical Trials Run 2 | cholecystectomy AND (bariatric surgery OR gastric bypass OR SADI OR OAGB OR duodenum OR roux-en-y) \| Active, not recruiting, Completed, Suspended, Terminated, Withdrawn, Unknown status |  |

| 1. **Should weight loss surgery versus standard medical management be used for treating NAFLD, NASH, or hepatic steatosis in morbidly obese patients?** | | |
| --- | --- | --- |
| **Database** | **Final search strategies** |  |
| PubMed Q2 | ((("Obesity"[mh] AND (morbid[tw] OR Morbidly[tw])) OR "Morbidly obese"[tw] OR "super-obese"[tw] OR "super obese"[tw]) AND (NASH [tw] OR NAFLD [tw] OR "hepatic steatosis"[TW] OR steatohepatitis [tw] OR "liver steatosis"[tw] OR "fatty liver"[mh]) AND ("medical treatment"[tw] OR "drug therapy"[sh] OR "therapeutic approach"[tw] OR "administration and dosage"[sh] OR "Anti-Obesity Agents"[mh] OR "Anti-Obesity Agents"[pa] OR "Diet, Food, and Nutrition"[mh] OR diet[tw] OR Dietary[tw] OR Conservative[tiab] OR exercise[mh] OR exercise[tw] OR "physical activity"[tw] OR "Life Style"[mh] OR "Pharmacologic treatment"[tw] OR "antagonists and inhibitors" [sh] OR antagonists[tw] OR "Bariatric Surgery"[mh] OR "bariatric surgery"[tw] OR "gastric bypass"[tw] OR Gastroenterostomy[mh] OR Roux-en-Y[tw] OR "Anastomosis, Roux-en-Y"[mh] OR OAGB[tw] OR "anastomosis gastric"[tw] OR duodenum/surgery[mh] OR duodenal switch[tw] OR SADI[tw] OR "single anastomosis"[tw] OR "gastric band"[tw] OR "gastric bands"[tw]) AND (Clinical Study[pt] OR Comparative Study[pt] OR "Epidemiologic Studies" [mh] OR Evaluation Study[pt] OR Meta-Analysis[pt] OR Multicenter Study[pt] OR Systematic Review[pt] OR randomized[tiab] OR study[tiab] OR studies [tiab] OR follow-up studies[mh] OR Practice Guideline[pt] OR "practice guidelines as topic"[mh] OR guideline[title] OR guidelines[title])) NOT ("gastric Balloon"[title] OR "intragastric dual Balloon"[title] OR "intragastric Balloon"[title] OR "intra-gastric Balloon"[title] OR "intragastric Balloons"[title] OR "Elipse Balloon"[title] OR Case reports[pt] OR "case report"[tw] OR ("animals"[MH:noexp] NOT "humans"[MH]) OR rat[tiab] OR rats[tiab] OR dog[tiab] OR dogs[tiab] OR porcine [tiab] OR "infant"[MeSH] OR (child[mh] NOT adult[mh]) OR children[title] OR child[title] OR infants[title] OR infant[title] OR pediatric [tiab] OR "1889:1989"[ppdat]) |  |
| Embase Q2 | (('fatty liver'/exp OR 'fatty liver' OR 'nonalcoholic steatohepatitis'/exp OR 'nonalcoholic steatohepatitis') AND ('morbid obesity'/exp OR 'morbid obesity') AND ([adult]/lim OR [aged]/lim OR [middle aged]/lim OR [very elderly]/lim OR [young adult]/lim) AND ('practice guideline'/exp OR 'practice guideline' OR [cochrane review]/lim OR [systematic review]/lim OR [meta analysis]/lim OR [clinical study]/lim)) NOT ([conference abstract]/lim OR 'case report'/exp OR 'case report' OR [animals]/lim OR [1889-1989]/py) |  |
| Cochrane Q2 | ("morbid obesity" OR "morbidly obese" OR "Super-obese") AND (NASH OR NAFLD OR "hepatic steatosis") |  |
| Clinical Trials Q2 | Morbid Obesity OR morbidly obese OR super-obese \| hepatic steatosis OR NASH OR NAFLD |  |
| PubMed | Above NOT ("1960/01/01"[CRDT] : "2020/07/20"[CRDT]) |  |
| Embase | Above NOT [1889-20196]/py |  |
| Cochrane | Above; with Cochrane Library publication date from Jun 2020 to Feb 2022 |  |
| Clinical Trials | Morbid Obesity OR morbidly obese OR super-obese \| Active, not recruiting, Completed, Suspended, Terminated, Withdrawn, Unknown status Studies \| hepatic steatosis OR NASH OR NAFLD |  |

| 1. **Should SG or intestinal bypass procedures be used in obese patients with inflammatory bowel disease (IBD)?** | | |
| --- | --- | --- |
| **Database** | **Final search strategies** |  |
| PubMed Q3 | (("Crohn’s disease" [tw] OR "Crohn disease" [tw] OR "Inflammatory Bowel Diseases"[mh] OR IBD[tiab] OR IBD [ot] OR "Ulcerative Colitis"[tw]) AND ("Bariatric Surgery "[mh] OR "bariatric surgery" [tw] OR "gastric bypass"[tw] OR Gastroenterostomy[mh] OR "Roux-en-Y"[tw] OR "Anastomosis, Roux-en-Y"[mh] OR OAGB[tw] OR "anastomosis gastric"[tw] OR duodenum/surgery[mh] OR "duodenal switch"[tw] OR SADI[tw] OR "single anastomosis"[tw] OR Gastroenterostomy[mh] OR "Sleeve gastrectomy"[tiab] OR "Sleeve gastrectomy" [ot] OR (Gastrectomy[mh] AND Sleeve [tw])) AND (Clinical Study[pt] OR Comparative Study[pt] OR "Epidemiologic Studies" [mh] OR Evaluation Study[pt] OR Meta-Analysis[pt] OR Multicenter Study[pt] OR Systematic Review[pt] OR randomized[tiab] OR study[tiab] OR studies [tiab] OR follow-up studies[mh] OR Practice Guideline[pt] OR "practice guidelines as topic"[mh] OR guideline[title] OR guidelines[title]) ) NOT ("Case reports"[pt] OR "case report"[tw] OR ("animals"[MH:noexp] NOT "humans"[MH]) OR rat[tiab] OR rats[tiab] OR dog[tiab] OR dogs[tiab] OR porcine[tiab] OR "infant"[MeSH] OR (child[mh] NOT adult[mh]) OR children[title] OR child[title] OR infants[title] OR infant[title] OR pediatric [tiab] OR "1965:1989" [ppdat]) |  |
| Embase  Q3 | ('ulcerative colitis'/exp OR 'ulcerative colitis' OR 'inflammatory bowel disease'/exp OR 'inflammatory bowel disease' OR 'crohn disease'/exp OR 'crohn disease') AND ('bariatric surgery'/exp OR 'bariatric surgery' OR 'roux en y' OR 'roux y anastomosis'/exp OR 'roux y anastomosis' OR 'roux-en-y gastric bypass'/exp OR 'roux-en-y gastric bypass' OR 'biliopancreatic bypass'/exp OR 'biliopancreatic bypass' OR 'duodenal switch' OR 'single anastomosis gastric bypass'/exp OR 'single anastomosis gastric bypass') AND ([adult]/lim OR [aged]/lim OR [middle aged]/lim OR [very elderly]/lim OR [young adult]/lim) AND ('practice guideline'/exp OR 'practice guideline' OR [cochrane review]/lim OR [systematic review]/lim OR [meta analysis]/lim OR [clinical study]/lim)) NOT ([conference abstract]/lim OR 'case report'/exp OR 'case report' OR [animals]/lim OR [1889-1989]/py) |  |
| Cochrane Q3 | (Crohn disease OR Ulcerative Colitis OR "Inflammatory Bowel Disease" OR IBD) AND ("bariatric surgery" OR "Gastric bypass" OR Roux-en-y OR SADI OR OAGB OR "Sleeve Gastrectomy") |  |
| Clinical Trials Q3 | (bariatric surgery OR gastric bypass OR SADI OR OAGB OR duodenum OR roux-en-y OR Sleeve Gastrectomy) \| Ulcerative Colitis OR Crohn Disease OR IBD |  |
| PubMed | Above NOT ("1960/01/01"[CRDT] : "2020/07/20"[CRDT]) |  |
| Embase | Above NOT [1889-2019]/py |  |
| Cochrane | Above; with Cochrane Library publication date from Jun 2020 to Feb 2022 |  |
| Clinical Trials | (bariatric surgery OR gastric bypass OR SADI OR OAGB OR duodenum OR roux-en-y OR Sleeve Gastrectomy) \| Active, not recruiting, Completed, Suspended, Terminated, Withdrawn, Unknown status Studies \| Ulcerative Colitis OR Crohn Disease OR IBD |  |

| 1. **In patients with GERD status post Laparoscopic Sleeve Gastrectomy (LSG) should surgical or medical therapy be used?** | | |
| --- | --- | --- |
| **Database** | **Final search strategies** |  |
| PubMed Q4 | (("gastroesophageal reflux"[mh] OR gerd[tw] OR Gastroesophageal Reflux[tw]) AND ("Sleeve gastrectomy"[tiab] OR "Sleeve gastrectomy" [ot] OR (Gastrectomy[mh] AND Sleeve [tw])) AND (Clinical Study[pt] OR Comparative Study[pt] OR "Epidemiologic Studies" [mh] OR Evaluation Study[pt] OR Meta-Analysis[pt] OR Multicenter Study[pt] OR Systematic Review[pt] OR randomized[tiab] OR study[tiab] OR studies [tiab] OR follow-up studies[mh] OR Practice Guideline[pt] OR "practice guidelines as topic"[mh] OR guideline[title] OR guidelines[title])) NOT ("Case reports"[pt] OR "case report"[tw] OR ("animals"[MH:noexp] NOT "humans"[MH]) OR rat[tiab] OR rats[tiab] OR dog[tiab] OR dogs[tiab] OR porcine[tiab] OR "infant"[MeSH] OR (child[mh] NOT adult[mh]) OR children[title] OR child[title] OR infants[title] OR infant[title] OR pediatric [tiab] OR "1965:1989" [ppdat]) |  |
| Embase  Q4 | (('gastroesophageal reflux'/exp OR 'gastroesophageal reflux') AND ('sleeve gastrectomy'/exp OR 'sleeve gastrectomy') AND ([adult]/lim OR [aged]/lim OR [middle aged]/lim OR [very elderly]/lim OR [young adult]/lim) AND ('practice guideline'/exp OR 'practice guideline' OR [cochrane review]/lim OR [systematic review]/lim OR [meta analysis]/lim OR [clinical study]/lim)) NOT ([conference abstract]/lim OR 'case report'/exp OR 'case report' OR [animals]/lim OR [1889-1989]/py) |  |
| Cochrane Q4 | (Sleeve gastrectomy) AND (GERD OR "gastroesophageal Reflux") |  |
| Clinical Trials Q4 | Sleeve Gastrectomy \| Gastroesophageal Reflux OR GERD |  |
| PubMed | Above NOT ("1960/01/01"[CRDT] : "2020/07/20"[CRDT]) |  |
| Embase | Above NOT [1889-2019]/py |  |
| Cochrane | Above with Cochrane Library publication date from Jun 2020 to Feb 2022 |  |
| Clinical Trials | Sleeve Gastrectomy \| Active, not recruiting, Completed, Suspended, Terminated, Withdrawn, Unknown status Studies \| Gastroesophageal Reflux OR GERD |  |
